# The current landscape of personalised preventive approaches for non-communicable diseases: A scoping review

**DOI:** 10.1371/journal.pone.0317379

**Published:** 2025-01-13

**Authors:** Sara Farina, Tommaso Osti, Luigi Russo, Alessandra Maio, Nicolò Scarsi, Cosimo Savoia, Abdelrahman Taha, Leonardo Villani, Roberta Pastorino, Stefania Boccia

**Affiliations:** 1 University Department of Life Science and Public Health, Section of Hygiene, Università Cattolica del Sacro Cuore, Rome, Italy; 2 Department of Woman and Child Health and Public Health, Fondazione Policlinico Universitario A. Gemelli IRCCS, Rome, Italy; Universita degli Studi di Roma Tor Vergata, ITALY

## Abstract

**Introduction:**

Personalised prevention offers a promising tool to reduce the impact of non-communicable diseases, which represent a growing health burden worldwide. However, to support the adoption of this innovation it is needed to clarify the current state of available evidence in this area. This work aims to provide an overview of recent publications on personalised prevention for chronic conditions.

**Materials and methods:**

A scoping review of scientific literature from Medline, Scopus, Web of Science and grey literature was conducted. Eligible articles included prospective primary studies and clinical practice directives on personalised preventive approaches for chronic diseases published between January 2017 to December 2023. The review followed Arksey-O’Malley guidelines and PRISMA-ScR checklist.

**Results:**

We identified 121 publications including 60 primary cohort studies and 61 clinical practice directives. We extracted 249 personalised preventive approaches, 27% in primary prevention, 27% in secondary prevention, and 46% in tertiary prevention. In primary prevention, 50% of the 67 approaches were from cohort studies, mainly targeting cardiovascular diseases, and 50% from directives primarily focused on cancer. Secondary prevention included 66 approaches, 73% from directives mainly concerning breast cancer. Tertiary prevention included 116 approaches, evenly distributed among the two publication types and focusing mostly on cancer and cardiovascular diseases. Lastly, tertiary prevention is the most represented level of prevention both in primary research studies and directives (54% and 41% respectively).

**Conclusions:**

Our study highlights a significant focus on personalised prevention in oncology in the past few years, with numerous recently issued clinical practice directives. We identified substantial original research in personalised primary prevention of cardiovascular diseases, indicating growing interest in the field. However, the distribution of primary studies and directives across the three preventive levels anticipate challenges in generating evidence of clinical utility in primary and secondary prevention, with most approaches falling under tertiary prevention.

## Introduction

According to the latest Global Burden of Disease Study, non-communicable diseases (NCDs) account for nearly 90% of deaths and over 80% of Disability-Adjusted Life Years in Europe [1]. These include cancer, cardiovascular diseases, diabetes, neurodegenerative and chronic respiratory conditions, and obesity which are common among individuals over 45 years old, often presenting with comorbidities [2]. The growing burden of these conditions raises concerns about the sustainability of healthcare systems. As multimorbidity becomes more complex, healthcare systems face increased strain, necessitating innovative strategies to maintain effective care [3]. Balancing prolonged care demands with the intricacies of multimorbidity highlights the urgent need for adaptable and comprehensive healthcare approaches. Prevention emerges as a crucial tool in reducing the burden of NCDs and supporting health system sustainability amidst evolving epidemiological challenges [4]. In response, personalised medicine has developed, aiming to provide optimal care to individuals at the right time by integrating data on lifestyle, metabolism, genetics, and social factors. This approach not only enhances health intervention effectiveness by tailoring it to individual characteristics but in principle it promotes resource optimisation, contributing to intervention sustainability [5].

Despite its initial distance from Population Health, personalised medicine converges with it in the concept of “Precision Public Health”. which scales personalised medicine to populations with a focus on prevention, leveraging genomic profiles and big data [6–8]. Evidence supporting the potential of personalised prevention to improve population health outcomes is substantial, and a number of research studies are ongoing, nevertheless its implementation in health systems remains limited [9, 10]. The great majority of the evidence available today in this field aims to assess the ability of these tests to predict specific genes or genetic variants (analytical validity) and their accuracy in predicting future clinical outcomes (clinical validity). This is an essential first step but demonstrating the clinical utility of a new technology—meaning its efficacy or effectiveness in improving patient health outcomes—is crucial for its adoption in national healthcare systems. Ideally, randomised controlled trials (RCTs) are the gold standard for evaluating the efficacy of personalised preventive approaches, although they can be challenging to conduct due to the rapid technological advancements that render such studies quickly outdated before completion, especially in primary and secondary prevention trials due to the length of time required to observe health outcomes [11, 12]. When a trial cannot be conducted, well designed observational studies can inform on the effectiveness of personalised preventive approaches. An example of widely implemented approaches in personalised primary and secondary prevention includes *BRCA1/2* testing for identifying individuals at risk for breast and gynaecologic cancers, that were informed from observational evidences [13]. More recently, emerging technologies like the Polygenic Risk Score (PRS) and other predictive tests show promise for identifying subgroups of population at higher risk of certain conditions, thus making them eligible to receive personalised preventive interventions. However, the evidence supporting the clinical effectiveness of these technologies is fragmented, hindering understanding of their readiness for scale-up in European health systems [14] based on accumulating evidences from RCTs and following clinical guidelines since more than a decade.

Our study aims to comprehensively map the current state of research efforts in personalised preventive approaches across the three levels of prevention by reviewing the recent scientific literature from prospective cohort studies including trials and observational evidence. Additionally, we aim to map the actual clinical practice directives including guidelines and recommendations that use personalised preventive approaches in order to compare the ongoing research efforts, with the actual implemented practice in personalised prevention.

## Materials and methods

This work is carried out in the context of the “a PeRsonalised Prevention roadmap for the future HEalThcare” (PROPHET) project, funded under the Horizon Europe programme [15]. According to PROPHET, personalised prevention is defined: *“Personalised prevention aims to prevent onset*, *progression and recurrence of disease through the adoption of targeted interventions that consider the biological information*, *environmental and behavioural characteristics*, *socio-economic and cultural context of individuals*. *This should be timely*, *effective and equitable in order to maintain the best possible balance in lifetime health trajectory”* [16].

The scoping review follows the 5-stage methodological framework described by Arksey and O’Malley [17]. The protocol has been uploaded to the Open Science Framework for public consultation, with registration DOI: https://doi.org/10.17605/OSF.IO/DBPHR.

### Preliminary research and definitions

We conducted a preliminary search on PubMed to define the search strategy and to identify eligibility criteria. According to PROPHET definition, “*A personalised preventive approach is an action*, *or a set of actions*, *in which the information provided by genetic and/or other omics biomarkers testing*, *combined with demographic*, *environmental and behavioural characteristics*, *socio-economic and cultural context of individuals*, *guides the decision-making process regarding one or more interventions aimed at preventing the onset*, *progression and recurrence of diseases*” [16]. In this context, the level of prevention is determined by the subsequent intervention following the test.

### Search strategy

We searched for any relevant publication related to personalised prevention approaches on PubMed, Scopus, Web of Science, Google Scholar, and on grey literature sources, including Google, guidelines repositories, websites of personalised medicine and genomics projects or consortia (e.g., the International Consortium for Personalised Medicine, ICPerMed), and relevant institutions (e.g., national and international public health institutions). Considering the launch of ICPerMed in November 2016 as a pivotal beginning for a number of research and implementation efforts in personalised medicine, the search was extended from January 2017 to December 2023.

The search strategy, tailored to each database, employed keywords related to three main concepts: “personalised prevention”, “approach”, and “omics”. Synonyms for chronic and common diseases were intentionally omitted from the search query to broaden the scope of results from scientific databases (S1 File).

### Eligibility criteria

In order to be eligible, the following criteria had to be fulfilled:

1. prospective primary studies (clinical trial or cohort study) that evaluated the clinical efficacy of a personalised preventive approach for NCDs;
2. clinical practice directives including guidelines and recommendations that featured a personalised preventive approach.

The reports from 1) and 2) had to include any common chronic condition [18], even those originating from rare genetic variants. Only studies published in English language were included.

Furthermore, literature reviews were initially included to conduct a backward citation screening in order to identify additional relevant publications.

Exclusion criteria encompassed pre-prints, study protocols, as well as studies solely exploring the clinical validity of genetic or other omics tests (e.g., genome-wide association studies, case-control studies, retrospective cohorts), simulation models studies, articles not involving omics sciences in prevention of common chronic diseases, and studies conducted on animals.

The documents identified were uploaded into the Rayyan software [19] and underwent a two-phase assessment to determine their eligibility, firstly as a screening through title and abstract, secondly by full text. The process of screening of the eligible documents and data extraction, described below, was piloted and conducted by independent researchers in order to facilitate the alignment among them and resolve potential disagreements.

### Data extraction

From each eligible document we extracted information about the first author, year of publication, type of publication, Country where the study was conducted or the clinical practice directives produced, disease/health condition, level of prevention (primary, secondary, tertiary), and the approach described.

Additionally, for each approach identified within the eligible documents, we extracted the following characteristics:

- category of omics test (e.g., genomic, pharmacogenomic, metabolomic);
- analytical details of the test (e.g., the specific gene analysed, the name of the genetic panel);
- the preventive intervention following the test (e.g., lifestyle interventions, target therapy, personalised screening).

### Data synthesis

To highlight which disease, test, and intervention have been prioritised in the scientific literature, and by expert panels or consortia in the recent years, we then grouped the eligible personalised preventive approaches according to: type of publication (primary studies or clinical practice directives), level of prevention, major health domains (cancer, cardiovascular diseases, metabolic conditions, neurological and psychiatric disorders, and others), disease type (e.g., cancer site, specific cardiovascular disease), the omics test used and the type of intervention following the test. Descriptive statistics were computed for each item. Results were presented narratively and delineated in tables.

### Reporting

The Preferred Reporting Items for Systematic reviews and Meta-Analyses extension for Scoping Reviews (PRISMA-ScR) Checklist was used for our review (S2 File).

## Results

**Fig 1:**
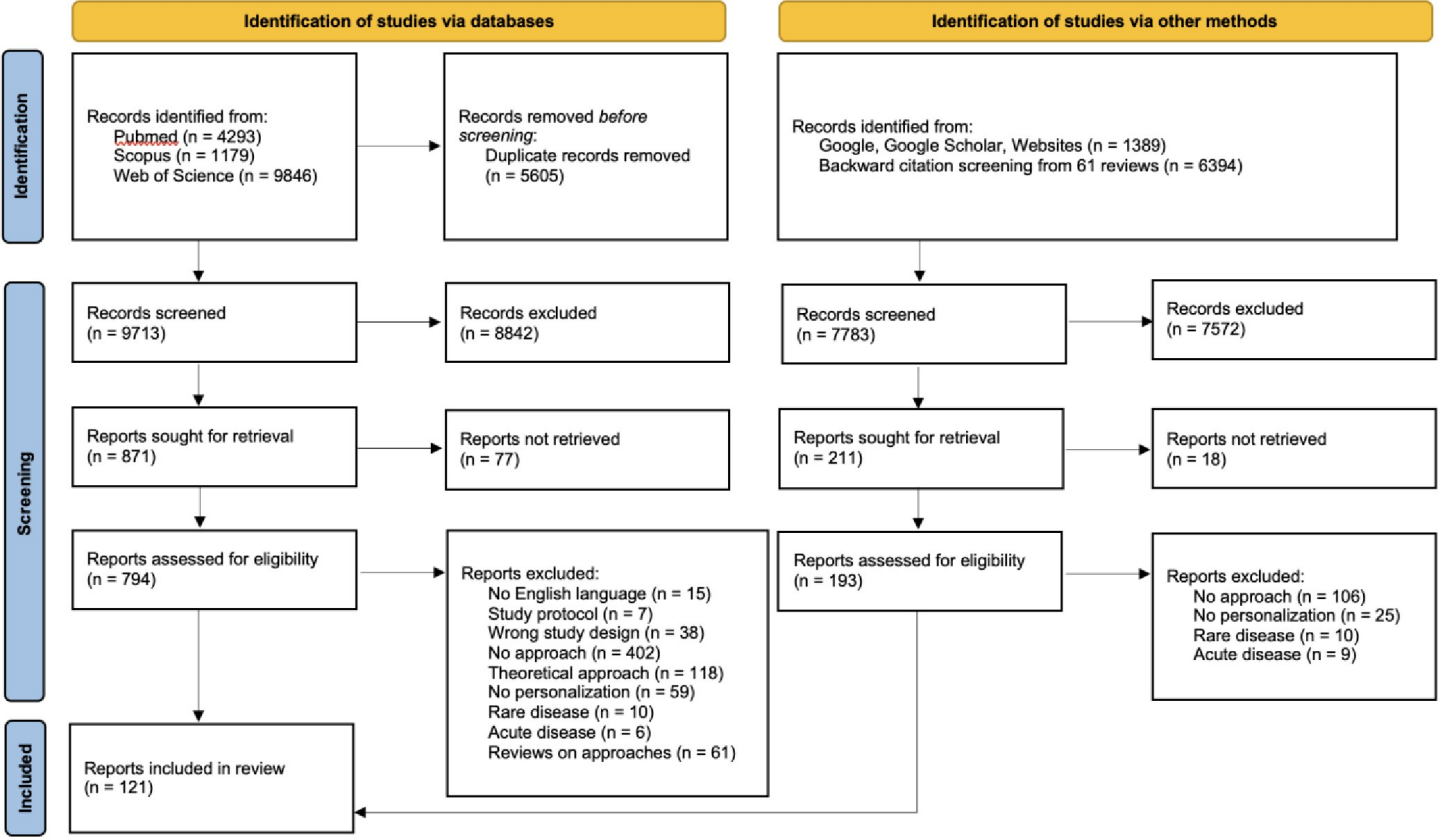
PRISMA flow chart for study selection.

A total of 121 records met the inclusion criteria, of which 78 were identified through scientific database searches and 43 from additional sources, as illustrated in the PRISMA flowchart (Fig 1). Fifty percent of the studies were conducted in the USA, 27% in Europe and 3% in Asia, while the remaining 16% consisted of multicentric studies or clinical practice directives from consortia of experts spanning various regions worldwide (S1 Table). Among the 121 studies included, 60 were primary cohort studies, comprising 46 clinical trials and 14 prospective observational studies, and 61 were clinical practice directives, including 47 guidelines and 14 recommendations.

**Fig 2:**
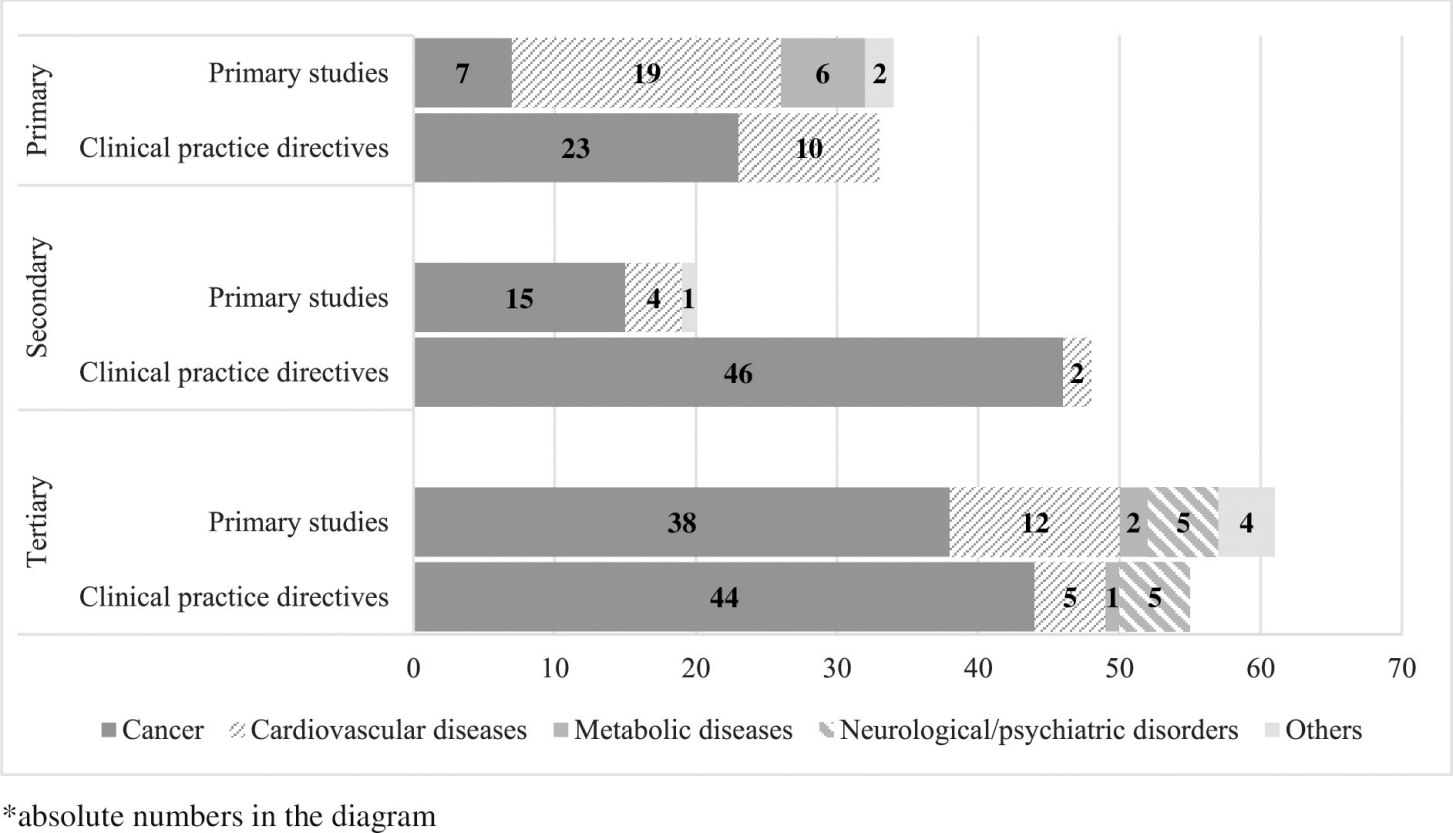
Details of the 249 personalised preventive approaches extracted from the 121 studies, according to the level of prevention, and classified by primary cohort studies (clinical trials or prospective observational studies) versus clinical practice directives (guidelines or recommendation), and by type of disease.

From the 121 eligible documents, a total of 249 approaches were identified that included a predictive omics test and a consequent preventive intervention, of which 113 from primary research studies (45%), and 136 from clinical practice directives (55%) (Fig 2).

According to the level of prevention, 67 (27%) were on primary prevention, 66 (27%) on secondary, and 116 (46%) on tertiary. Among primary preventive approaches, 51% were from primary studies and 49% from clinical practice directives, among secondary 27% were from primary studies and 73% from clinical practice directives, while for tertiary 53% were from primary studies and 47% from clinical practice directives.

Table 1 details the mapping results of the 249 personalised preventive approaches according to the level of prevention, the study design and the type of disease.

**Table 1:** Personalised preventive approaches by level of prevention, study design and type of disease.

	Primary	Secondary	Tertiary	Total of approaches
	prevention	prevention	prevention	
***Primary cohort studies***	**34 (30%)**	**18 (16%)**	**61 (54%)**	**113 (100%)**
**Clinical trials (N = 46)**	25 (36%)	3 (4%)	41 (60%)	69 (100%)
Cancer	3	2	19	24
Cardiovascular diseases	15	1	11	27
Metabolic conditions	6	-	2	8
Neurological/psychiatric disorders	-	-	5	5
Others	1	-	4	5
**Prospective observational studies (N = 14)**	9 (21%)	15 (34%)	20 (45%)	44 (100%)
Cancer	4	12	19	35
Cardiovascular diseases	4	2	1	7
Metabolic conditions	-	-	-	-
Neurological/psychiatric disorders	-	-	-	-
Others	1	1	-	2
***Clinical practice directives***	**33 (24%)**	**48 (35%)**	**55 (41%)**	**136 (100%)**
**Guidelines (N = 47)**	15 (19%)	22 (27%)	43 (54%)	80 (100%)
Cancer	10	22	35	67
Cardiovascular diseases	5	-	3	8
Metabolic conditions	-	-	1	1
Neurological/psychiatric disorders	-	-	4	4
Others	-	-	-	-
**Recommendations (N = 14)**	18 (32%)	26 (46%)	12 (22%)	56 (100%)
Cancer	13	24	9	46
Cardiovascular diseases	5	2	2	9
Metabolic conditions	-	-	-	-
Neurological/psychiatric disorders	-	-	1	1
Others	-	-	-	-
**Total of included studies (N = 121)**	**67 (27%)**	**66 (27%)**	**116 (*46*%)**	**249 (100%)**

One hundred-thirteen approaches were mapped among primary research studies, of which the vast majority were in the tertiary prevention setting (54%), then primary (30%) and secondary prevention (16%). Sixty-nine approaches were mapped from clinical trials, of which the vast majority was in the setting of tertiary prevention (61%), especially on cancer, followed by primary and secondary preventive studies in the setting of cardiovascular diseases. Observational studies included 44 approaches, of which the highest proportion (45%) was in tertiary prevention, with cancer being the most common, and secondary cancer prevention.

As for the clinical practice directives, we mapped 136 approaches, of which the majority were in the tertiary prevention setting (41%), followed by secondary (35%) and primary (24%). Among them, 80 different approaches were mapped from guidelines, of which the majority was in the setting of tertiary prevention (54%), especially on cancer, followed by primary and tertiary preventive studies. Lastly, recommendations included 56 approaches, of which the highest proportion (46%) was in secondary prevention, with cancer being the most common, and primary cancer prevention.

The 121 records included in the review and the 249 approaches extracted are listed in the S2 Table and detailed in the following paragraphs, according to the level of prevention.

### Primary prevention

A total of 67 approaches on personalised primary prevention were identified, including 34 from primary cohort studies and 33 from clinical practice directives.

#### Primary cohort studies

The 34 approaches focused on cardiovascular diseases (56%), cancer (21%), metabolic conditions (18%), and other chronic diseases (5%) (Table 2), of which 25 were from clinical trials and 9 from prospective observational studies (Table 1). The vast majority of the approaches investigated the genetic predisposition and pharmacogenomic profile for high-risk individuals followed by the administration of personalised risk-reduction strategies for coronary heart disease, and for breast, ovarian and pancreatic cancers. Four approaches in cancer (melanoma and breast cancer) and cardiovascular diseases included PRSs profiling and disclosure among high-risk subjects in order to encourage the adoption of healthier lifestyles or the uptake of other preventive therapies.

**Table 2:** Personalised approaches in primary prevention (N = 67) according to disease category, specific health condition, test evaluated and consequent preventive intervention.

Disease	Specific condition	N (%)	Test	N (%)	Intervention	N (%)
**Primary cohort studies (N = 34)**						
**Cancer** N = 7 (21%)	Breast cancer	3 (42%)	Multigene panels	3 (42%)	Prophylactic surgery	3 (42%)
	Melanoma	2 (28%)	Monogenic tests	2 (28%)	Lifestyle changes	3 (42%)
	Ovarian cancer	1 (15%)	Polygenic risk score	2 (28%)	Preventive therapy	1 (16%)
	Pancreatic cancer	1 (15%)				
**Cardiovascular diseases** N = 19 (56%)	Coronary artery diseases	18 (95%)	Genetic tests	14 (74%)	Drug dose adaptation	13 (68%)
	*- Familial hypercholesterolemia*	*11 (58%)*	Pharmacogenomic tests	3 (16%)	Lifestyle changes	6 (32%)
	*- Hypertension*	*2 (10%)*	Polygenic risk score	2 (10%)		
	Dilated cardiomyopathy	1 (5%)				
**Metabolic diseases** N = 6 (18%)	Type 2 diabetes	4 (67%)	Genetic tests	5 (83%)	Lifestyle changes	6 (100%)
	Obesity	2 (33%)	Nutrigenomic test	1 (17%)		
**Others** N = 2 (5%)	Eye macular degeneration	1 (50%)	Genetic tests	2 (100%)	Lifestyle changes	2 (100%)
	Rheumatoid arthritis	1 (50%)				
**Clinical practice directives (N = 33)**						
**Cancer** N = 23 (70%)	Breast cancer	9 (39%)	Monogenic tests	15 (62%)	Prophylactic surgery	16 (70%)
	Ovarian cancer	7 (33%)	Multigene panels	8 (38%)	Lifestyle changes	4 (17%)
	Colorectal cancer	3 (13%)			Preventive therapy	2 (3%)
	Pancreatic cancer	2 (9%)				
	Uterine cancer	1 (3%)				
	Prostatic cancer	1 (3%)				
**Cardiovascular diseases** N = 10 (30%)	Coronary artery diseases	10 (100%)	Genetic tests	8 (80%)	Drug dose adaptation	7 (70%)
	*- Familial hypercholesterolemia*	*7 (70%)*	Pharmacogenomic tests	2 (20%)	Lifestyle changes	3 (30%)
	*- Hypercholesterolemia*	*1 (10%)*				
	*- Thromboembolism*	*1 (10%)*				
	*- Channelopathies*	*1 (10%)*				

Concerning the approaches on metabolic conditions, four were on diabetes and two on obesity, while the strategies on other chronic diseases were specifically on rheumatoid arthritis and age-related macular degeneration; these approaches were based on the use of genetic testing for the identification of high-risk subjects and target life-style interventions.

#### Clinical practice directives

Thirty-three approaches were included, of which 15 were from guidelines and 18 from recommendations (Table 1). Among them, 70% focused on cancer and 30% on cardiovascular diseases. Among cancer prevention approaches, breast cancer (39%) and ovarian cancer (30%) were the most common, followed by colorectal, pancreatic, uterine and prostatic cancer (31%). These approaches included cascade genetic screening of *BRCA1/2* mutation testing, and other hereditary syndromes, which targeted healthy individuals with family history or other risk factors. In these situations, individuals identified as being at high risk through the tests, preventive interventions included risk-reduction strategies, of which 70% included prophylactic surgery, 17% promotion of healthy behaviours, and 13% preventive treatments, such as chemoprophylaxis. In the cardiovascular disease setting, interventions such as lifestyle modification and the use of preventive medications were included in 30% and 70% of the approaches aimed at preventing coronary artery diseases. Such strategies included genetic tests for familial hypercholesterolemia (70%), channelopathies (10%), and pharmacogenomic tests (20%), enabling personalised therapies and adverse reactions prevention.

### Secondary prevention

The review identified 66 approaches for personalised secondary prevention, comprising 18 from primary cohort studies and 48 from clinical practice directives (Table 3).

**Table 3:** Personalised approaches in secondary prevention (N = 66) according to disease category, specific health condition, test evaluated and consequent preventive intervention.

Disease	Specific conditions	N (%)	Test	N (%)	Intervention	N (%)
**Primary studies (N = 18)**						
**Cancer** N = 14 (78%)	Breast cancer	3 (22%)	Multigene panels	11 (79%)	Personalised cancer screening	14 (100%)
	Pancreatic cancer	2 (14%)	Monogenic tests	3 (21%)		
	Colorectal cancer	2 (14%)				
	Prostatic cancer	2 (14%)				
	Other cancers	5 (36%)				
**Cardiovascular diseases** N = 3 (17%)	Coronary artery diseases	2 (67%)	Genetic tests	2 (67%)	Personalised follow-up	3 (100%)
	*- Familial hypercholesterolemia*	*2 (67%)*	Whole genome sequencing	1 (33%)		
	Hypertrophic cardiomyopathy	1 (33%)				
**Others** N = 1 (5%)	Eye macular degeneration	1 (100%)	Genetic tests	1 (100%)	Personalised follow-up	1 (100%)
**Clinical practice directives (N = 48)**						
**Cancer** N = 46 (96%)	Breast cancer	14 (30%)	Monogenic tests	29 (63%)	Personalised cancer screening	46 (100%)
	Colorectal cancer	10 (22%)	Multigene panels	17 (37%)		
	Pancreatic cancer	6 (13%)				
	Ovarian cancer	3 (6%)				
	Renal cancer	2 (5%)				
	Other cancers	11 (24%)				
**Cardiovascular diseases** N = 2 (4%)	Coronary artery diseases	1 (50%)	Genetic tests	2 (100%)	Personalised follow-up	2 (100%)
	*- Familial hypercholesterolemia*	*1 (50%)*				
	Cardiomyopathies	1 (10%)				

#### Primary cohort studies

The 18 approaches focused on cancer (78%), cardiovascular diseases (17%), and eye macular degeneration (5%) (Table 3), of which 3 were from clinical trials and 15 from observational studies (Table 1). In secondary cancer prevention, the majority of the approaches included cascade genetic screening for breast cancer (22%), while a lower number focused on colorectal, prostate, and pancreatic cancer (14% each) and other tumours (36%). After identifying at-risk individuals through genetic counselling and testing, the approaches included personalised screening strategies aimed at early cancer detection.

Regarding cardiovascular diseases, two approaches focused on familial hypercholesterolemia diagnosis based on genetic testing, and follow-up to prevent coronary artery disease; and one approach aimed at identifying high risk individuals with hypertrophic cardiomyopathy genetic testing to target precise cardiological follow-up.

#### Clinical practice directives

Forty-eight approaches were included, of which 22 were from guidelines and 26 from recommendations (Table 1). Among them, 96% focused on cancer, while only 4% pertained to cardiovascular diseases.

Among cancer, 30% aimed to enhance breast cancer screening programmes, including magnetic resonance imaging for high-risk individuals with *BRCA1/2* mutations or other associated genetic variants. This same strategy, which includes cascade genetic screening and subsequent personalised follow-up, was also present in approaches for colorectal cancer (22%), pancreatic cancer (13%), ovarian cancer (6%), renal cancer (5%), and other cancers (24%), including endocrine tumours, melanoma, prostate cancer, and others. Similarly for primary prevention, genetic panels analysis for various hereditary conditions were present in 37% of the approaches. Regarding cardiovascular diseases, one approach focused on coronary artery disease, including familial hypercholesterolaemia genetic testing, and the other on cardiomyopathies genetic variants detection, to enable personalised follow-up through periodic cardiological visits and imaging.

### Tertiary prevention

The review identified 116 approaches, comprising 61 from primary cohort studies and 55 from clinical practice directives (Table 4).

**Table 4:** Personalised approaches in tertiary prevention (N = 116) according to disease category, specific health condition, test evaluated and consequent preventive intervention.

Disease	Specific conditions	N (%)	Test	N (%)	Intervention	N (%)
**Primary cohort studies (N = 61)**						
**Cancer** N = 38 (62%)	Colorectal cancer	8 (22%)	Multigene panels	26 (68%)	Target therapy	36 (95%)
	Breast cancer	7 (18%)	Monogenic tests	10 (27%)	Drug dose adaptation	2 (5%)
	Lung cancer	7 (18%)	Pharmacogenomic tests	2 (5%)		
	Ovarian cancer	5 (13%)				
	Pancreatic cancer	3 (8%)				
	Prostatic cancer	2 (5%)				
	Cholangiocarcinoma	2 (5%)				
	Other cancers	4 (11%)				
**Cardiovascular diseases** N = 12 (20%)	Coronary artery diseases	11 (92%)	Pharmacogenomic tests	12 (100%)	Drug dose adaptation	12 (100%)
	Atrial fibrillation	1 (8%)				
**Metabolic diseases** N = 2 (3%)	Type 2 diabetes	1 (50%)	Pharmacogenomic tests	2 (100%)	Drug dose adaptation	2 (100%)
	Obesity	1 (50%)				
**Neurological and psychiatric disordes** N = 5 (8%)	Depression disorders	2 (40%)	Pharmacogenomic tests	5 (100%)	Drug dose adaptation	5 (100%)
	Epilepsy	1 (20%)				
	Dementia	1 (20%)				
	Alzheimer	1 (20%)				
**Others** N = 4 (7%)	Eye macular degeneration	1 (25%)	Pharmacogenomic tests	4 (100%)	Drug dose adaptation	4 (100%)
	Chronic obstructive pulmonary disease	1 (25%)				
	Gastroesophageal reflux	1 (25%)				
	Hypothyroidism	1 (25%)				
**Clinical practice directives (N = 55)**						
**Cancer** N = 44 (80%)	Breast cancer	18 (41%)	Multigene panels	22 (50%)	Target therapy	37 (84%)
	Colorectal cancer	5 (12%)	Monogenic tests	15 (34%)	Drug dose adaptation	7 (16%)
	Ovarian cancer	4 (9%)	Pharmacogenomic tests	7 (16%)		
	Lung cancer	3 (7%)				
	Prostatic cancer	3 (7%)				
	Melanoma	2 (4%)				
	Gastric cancer	2 (4%)				
	Pancreatic cancer	2 (4%)				
	Other cancers	5 (12%)				
**Cardiovascular diseases** N = 5 (9%)	Coronary artery diseases	3 (60%)	Pharmacogenomic tests	5 (100%)	Drug dose adaptation	5 (100%)
	Cardiomyopathies	1 (20%)				
	Atrial fibrillation	1 (20%)				
**Metabolic diseases** N = 1 (2%)	Gout	1 (100%)	Pharmacogenomic tests	1 (100%)	Drug dose adaptation	1 (100%)
**Neurological and psychiatric disordes** N = 5 (9%)	Depression disorders	2 (40%)	Pharmacogenomic tests	5 (100%)	Drug dose adaptation	5 (100%)
	Epilepsy	2 (40%)				
	Schizophrenia	1 (20%)				

#### Primary cohort studies

The 61 approaches focused on cancer (62%), cardiovascular diseases (20%), and eye macular degeneration (5%), neurological and psychiatric diseases (8%), metabolic diseases (3%), and 7% on other chronic conditions (Table 4); among them, 41 were from clinical trials and 20 from observational studies (Table 1).

For cancer prevention 22% of approaches focused on colorectal cancer, followed by breast (18%) and lung cancer (18%). The approaches identified included the use of multigene panels (68%) and single gene mutation tests (27%) to identify target therapies, and pharmacogenomic tests (5%) to determine the appropriate dosage of anticancer drugs. In cardiovascular disease approaches, 92% focused on pharmacogenomics for myocardial infarction maintenance and 8% on personalised treatment of atrial fibrillation. Regarding neurological and psychiatric diseases, pharmacogenomics was used in five approaches: two for depressive disorders, and one on epilepsy, dementia, and Alzheimer’s disease. In metabolic diseases, pharmacogenomic approaches were used in one study on the treatment of type 2 diabetes and one on obesity.

Finally, four approaches were identified from a randomised controlled trial evaluating the pharmacogenomic profile of patients undergoing polypharmacotherapy for common chronic diseases, including eye macular degeneration, chronic obstructive pulmonary disease, gastroesophageal reflux disease, and hypothyroidism (Table 4).

#### Clinical practice directives

Fifty-five tertiary prevention approaches were identified from clinical practice directives, 43 from guidelines and 12 from recommendations (Table 1). Consistent with findings in primary and secondary prevention, the majority pertained to cancer (80%), followed by cardiovascular diseases (9%), neurological and psychiatric conditions (9%), and metabolic diseases. For tertiary prevention of cancer, the identified approaches primarily included the detection of somatic mutations. These tests may screen for a range of genetic variants using multigene panels (50%), or alternatively, they may focus on specific gene variants, such as *BRCA1*, *BRCA2*, and *BRAF* (34%), to guide and target therapies. Another strategy involved pharmacogenomic tests (16%), particularly genotyping *DPYD*, *UGT1A*, and *CYP2D6* genes, to ensure proper dosing of anticancer drugs like dihydropyridines, irinotecan, and tamoxifen. These approaches were identified for various cancers, with the majority focused on breast cancer, followed by colorectal, ovarian and lung cancer (Table 4).

Regarding cardiovascular diseases, three approaches focused on coronary artery disease, including pharmacogenomic tests for *CYP2C19* genotyping, which is implicated in the metabolism of antiplatelet drugs, to establish correct maintenance therapy dosages for patients who had undergone percutaneous coronary intervention for myocardial infarction. The remaining two approaches, also based on pharmacogenomic testing, focused on preventing complications and personalising treatment for cardiomyopathies and atrial fibrillation to prevent events.

For neurological and psychiatric diseases, the approaches centred on pharmacogenomic tests to determine the correct dosage of drugs for treating conditions, including depressive disorders (40%), epilepsy (40%), and schizophrenia (20%). Lastly, one approach for metabolic diseases focused on gout treatment, through appropriate therapy and dosage to prevent complications, including renal insufficiency and treatment-related events.

## Discussion

This scoping review provides a broad overview of publications and reports on personalised preventive approaches for chronic diseases published in the recent years, both from primary research studies including trials and observational studies, and clinical practice directives. While primary studies reflect current research advancements, recommendations and guidelines indicate well-established approaches with longstanding evidence. The results from our study show that half of the outputs were published in the US, with a good balance between the two main kind of documents. When extracting the approaches from the reports, around half concerned primary research studies, with a larger proportion of clinical trials respect to observational studies, and half clinical practice directives, thus suggesting an important amount of evidence accumulated from primary studies in the past decades in the field. As for the level of prevention, most of the personalised approaches were on tertiary prevention (46%) compared to primary (27%) and secondary (27%), with secondary prevention showing a greater proportion of outputs from clinical practice directives respect to primary studies. As for the disease investigated, cancer was the most common across the studies and the preventive level, with exception of CVD that are well represented among clinical trials.

Indeed, deepening the analysis of our results through the comparison between clinical practice directives and primary studies, different maturity levels of evidence in personalised prevention emerge. When examining the diseases targeted, the wide representation of oncology, which accounts for 69% of the approaches, reflects the significant interest the scientific community has dedicated to research in this field over recent decades. For all levels of prevention in oncology, there are more clinical practice directives than primary studies, especially in primary and secondary prevention. This trend differs from other diseases and can be attributed to the extensive research and established strategies in cancer prevention prior to our selected timeframe. In fact, while details like recommended screening ages or lifestyle advice may vary, the guidelines largely use the same genetic tests. A paramount example in this context are breast and ovarian cancer, which have emerged as one of the most central conditions for which these approaches are designed across primary and secondary preventive settings. The clinical practice directives included for breast and ovarian cancer prevention largely rely on the same genetic tests, (*BRCA1/2)*, *STK11*, *TP53*, and differed in aspects like the recommended target age, or specific intervention details.

Concerning CVD, a growing interest is testified by a large number of primary research studies across the three levels of prevention. These include the use of PRS to trigger improved lifestyles for primary prevention, for example by evaluating how the return of risk class assessed by PRS integration influences the adoption of virtuous behaviours in high-risk individuals [20]. In terms of secondary prevention, we identified innovative approaches for personalised screening programmes towards people with predisposition for familial hypercholesterolemia [21]. As for tertiary prevention, the majority of primary studies is represented by example of pharmacogenomics testing to adapt therapies to prevent the recurrence of major CVD events like *CYP2C19* genotyping to implement *P2Y12* inhibitor dose adjustments [22–24]. Clinical practice directives, instead, primarily concentrate on managing familial hypercholesterolemia, with guidelines differing in the target population and the recommended interventions, which are personalised based on the patient’s individual characteristics and the clinical context [25, 26]. The outlook for neurological, psychiatric, and metabolic diseases is instead rather bleak, with scarce outputs published, which are still firmly anchored to primary studies and tertiary prevention. This limited focus is concerning, especially considering the increasing ageing population and the associated burden of disease among the oldest-old, which includes particularly neurodegenerative disorders such as dementia [27]. This may partly reflect the challenges associated with these conditions, including the complex interplay of genetic and environmental factors and the need for long-term studies to establish effective preventive measures, as well as the barriers in securing research funding in a field often less influenced by for-profit incentives [28]. Pharmacogenomics, however, is the only approach that consistently emerges in guidelines for the treatments of these diseases, playing a pioneering role in translating personalised prevention from research to clinical practice. It acts as a catalyst for primary research in tertiary prevention, showcasing its significant impact on improving therapies for several non-communicable diseases. This model serves as an exemplary framework that could be replicated in various contexts or approaches, emphasising the potential of genetic insights to enhance individualised healthcare strategies. By integrating pharmacogenomic data, clinicians can better tailor prevention and treatment plans, ultimately leading to more effective health outcomes [29].

Overall, our study reveals that the promising evidence in the area of personalised prevention struggles to find application in clinical practice, or even earlier, in producing solid evidence of the clinical utility of tools with established predictive power. This is because translating predictive omics evidence into clinical practice involves several challenges [30–32]. Producing evidence for primary and secondary prevention is particularly difficult and costly, requiring rigorous clinical studies with long-term follow-up [33]. The extended timeframes and significant resources needed for such studies often hinder the generation of robust evidence, slowing the translation of research findings into clinical practice. Moreover, even when solid evidence is obtained, multidisciplinary evaluations are needed to complement recommendations with considerations on organisational feasibility, resource sustainability, equitable access to care, and acceptability among both the target population and healthcare professionals [34, 35]. Although our study did not address the assessment of these domains in the included studies, the scarcity of literature specifically focusing on these aspects, particularly including equity and equality, remains evident [36, 37]. These evaluations are crucial for ensuring that new preventive measures can be effectively integrated into existing healthcare systems and are accessible to all patients who may benefit from them.

The complexity of these evaluations is compounded by the need to balance the potential benefits of personalised prevention with the practical constraints of healthcare delivery. For instance, integrating omics data into routine clinical practice requires significant investment in infrastructure, training for healthcare professionals, and patient education. Additionally, ethical considerations, such as patient privacy and informed consent must be carefully managed to build trust and ensure the responsible use of genetic information.

Nonetheless, European countries are making substantial advances through initiatives such as Genomics England [38], the Estonian Biobank [39], FinnGen in Finland [40], and the IMPACT cohort in Spain [41]. These large-scale cohort studies systematically integrate genomic data with additional health and lifestyle metrics, creating invaluable resources for advancing research and facilitating the future implementation of personalised prevention strategies across Europe. Our study has several limitations. Firstly, as a scoping review, its objective is to explore the breadth and nature of personalised prevention, which means it does not assess the quality of the included studies or provide a critical synthesis of the results to evaluate their effectiveness. Secondly, the review may not be exhaustive of all existing approaches, leaving some potentially valuable strategies unexamined. Additionally, the eligibility criteria limited the results to records published after 2016 and those in English, potentially omitting relevant research. Lastly, our work does not evaluate the actual implementation and integration of the identified approaches into clinical practice.

In conclusion, our study highlights significant progress and ongoing challenges in personalised prevention. While oncology leads the way with broader guidelines and more robust evidence, other fields lag behind, necessitating further research and multidisciplinary efforts to translate omics data into clinical practice and develop inclusive, effective prevention strategies. The journey from evidence to implementation is complex, but the potential benefits for public health are substantial. As personalised medicine continues to evolve, ongoing collaboration among researchers, clinicians, policymakers, and patients will be essential to realise its full potential in improving health outcomes and preventing disease.

## Supporting information

S1 File Search strategies for scientific databases and grey literature. (DOCX)

S2 File Preferred Reporting Items for Systematic reviews and Meta-Analyses extension for Scoping Reviews (PRISMA-ScR) checklist. (DOCX)

S1 Table Countries where the studies were conducted or the clinical practice directives produced. (DOCX)

S2 Table Detailed overview of included approaches. (DOCX)
