# Supplementary material for: The current landscape of personalised preventive approaches for non-communicable diseases: A scoping review
Source: PLoS One. 2025 Jan 13;20(1):e0317379. doi: 10.1371/journal.pone.0317379 (PMC11729939; doi:10.1371/journal.pone.0317379)
Supplement: S1 Table — (DOCX) [file pone.0317379.s003.docx]

**S1 Table. Countries where the studies were conducted or the clinical practice directives produced.**

| **Country** | **Clinical practice directives** | **Primary cohort studies** | **Total** |
| --- | --- | --- | --- |
| USA | 37 | 25 | 62 (50%) |
| Europe | 20 | 13 | 33 (27%) |
| *Multi-countries* | *9* | *2* | *11 (9%)* |
| *Italy* | *2* | *3* | *5 (4%)* |
| *The Netherlands* | *4* | *1* | *5 (4%)* |
| *Estonia* | *-* | *3* | *3 (2%)* |
| *Germany* | *2* | *1* | *3 (3%)* |
| *Denmark* | *1* | *-* | *1 (1%)* |
| *Croatia* | *1* | *-* | *1 (1%)* |
| *Finland* | *-* | *1* | *1 (1%)* |
| *France* | *-* | *1* | *1 (1%)* |
| *Spain* | *-* | *1* | *1 (1%)* |
| *UK* | *1* | *-* | *1 (1%)* |
| Asia | 1 | 2 | 3 (3%) |
| *China* | *-* | *1* | *1 (1%)* |
| *Japan* | *1* | *-* | *1 (1%)* |
| *Israel* | *-* | *1* | *1 (1%)* |
| Canada | - | 3 | 3 (3%) |
| Australia | - | 1 | 1 (1%) |
| Multi-countries | 3 | 16 | 19 (16%) |
| **Total** | **61** | **60** | **121 (100%)** |
