# Supplementary material for: The current landscape of personalised preventive approaches for non-communicable diseases: A scoping review
Source: PLoS One. 2025 Jan 13;20(1):e0317379. doi: 10.1371/journal.pone.0317379 (PMC11729939; doi:10.1371/journal.pone.0317379)
Supplement: S2 Table — (DOCX) [file pone.0317379.s004.docx]

**S2 Table. Detailed overview of included approaches.**

| **First Author, year (ref)** | **Study design** | **General disease** | **Specific disease** | **Prevention level** | **Omic science** | **Test** | **Intervention** |
| --- | --- | --- | --- | --- | --- | --- | --- |
| Abraham, 2023 (1) | Guidelines | Cancer | Breast | Tertiary | Genetics | HR/HER2, BRCA1/2, PALB2, PIK3CA, ESR1, NTRK, MSI-H, RET, TMB-H testing | Target therapy |
| Abu-Rustum, 2024 (2) | Guidelines | Cancer | Cervical | Tertiary | Genetics | PDL-1 testing | Target therapy |
| Abu-Rustum, 2024 (3) | Guidelines | Cancer | Uterine | Tertiary | Genetics | MMR IHC, MSI testing | Target therapy |
| Aguirre, 2018 (4) | Cohort study | Cancer | Pancreatic | Secondary | Genetics | Germline mutations detections in advanced pancreatic cancer patients and genetic counselling and screening for healthy relatives | Personalised screening |
| Ajani, 2022 (5) | Guidelines | Cancer | Gastric | Tertiary | Genetics | HER2, PD-L1, MSI/MMR testing | Target therapy |
| Alonso, 2021 (6) | Clinical trial | CVD | MACE (FH) | Primary | Genetics | FH genetic testing | Preventive therapy |
| Alver, 2018 (7) | Cohort study | CVD | MACE (FH) | Primary | Genetics | PCSK9, APOB, LDLR testing | Lifestyle changes |
| Alver, 2018 (7) | Cohort study | CVD | MACE (FH) | Primary | Genetics | PCSK9, APOB, LDLR testing | Preventive therapy |
| Alver, 2018 (7) | Cohort study | CVD | MACE (FH) | Secondary | Genetics | PCSK9, APOB, LDLR testing | Genetic counselling and personalised screening of healthy relatives |
| Amstutz, 2017 (8) | Guidelines | Cancer | Colorectal | Tertiary | Pharmacogenomics | DPYD genotyping | Personalised therapy |
| Andre, 2019 (9) | Clinical trial | Cancer | Breast | Tertiary | Genomics | Oncotype DX | Target therapy |
| Armstrong, 2024 (10) | Guidelines | Cancer | Ovarian | Tertiary | Genetics | BRCA1/2 testing | Target therapy |
| Benson, 2024 (11) | Guidelines | Cancer | Colorectal | Tertiary | Genetics | RAS and BRAF mutations; HER2 amplifications; MMR or MSI status (if not previously done) – Testing should be conducted as part of broad molecular profiling, which would identify rare and actionable mutations and fusions such as POLE/ POLD1, RET, and NTRK. | Target therapy |
| Beunk, 2024 (12) | Recommendations | Neuropsychiatric disorder | Schizophrenia | Tertiary | Pharmacogenomics | CYP2D6, CYP3A4 genotyping | Personalised therapy |
| Binderup, 2022 (13) | Guidelines | Cancer | Retinal hemangioblastoma | Secondary | Genetics | VHL testing | Personalised screening |
| Binderup, 2022 (13) | Guidelines | Cancer | Pancreatic | Secondary | Genetics | VHL testing | Personalised screening |
| Binderup, 2022 (13) | Guidelines | Cancer | Renal | Secondary | Genetics | VHL testing | Personalised screening |
| Birnbaum, 2021 (14) | Cohort study | CVD | MACE (FH) | Primary | Genetics | FH genetic testing | Preventive therapy |
| Birnbaum, 2021 (14) | Cohort study | CVD | MACE (FH) | Secondary | Genetics | FH genetic testing | Genetic counselling and personalised screening of healthy relatives |
| Boland, 2022 (15) | Recommendations | Cancer | Colorectal | Primary | Genetics | Peutz-Jeghers Syndrome genetic testing | Prophylactic surgery |
| Boland, 2022 (15) | Recommendations | Cancer | Colorectal | Secondary | Genetics | Peutz-Jeghers Syndrome genetic testing | Personalised screening |
| Boland, 2022 (15) | Recommendations | Cancer | Breast | Secondary | Genetics | Peutz-Jeghers Syndrome genetic testing | Personalised screening |
| Boland, 2022 (15) | Recommendations | Cancer | Pancreatic | Secondary | Genetics | Peutz-Jeghers Syndrome genetic testing | Personalised screening |
| Boland, 2022 (15) | Recommendations | Cancer | Ovarian | Secondary | Genetics | Peutz-Jeghers Syndrome genetic testing | Personalised screening |
| Boland, 2022 (15) | Recommendations | Cancer | Testicular | Secondary | Genetics | Peutz-Jeghers Syndrome genetic testing | Personalised screening |
| Boland, 2022 (15) | Recommendations | Cancer | Colorectal | Primary | Genetics | Juvenile Polyposis Syndrome genetic testing | Prophylactic surgery |
| Boland, 2022 (15) | Recommendations | Cancer | Colorectal | Secondary | Genetics | Juvenile Polyposis Syndrome genetic testing | Personalised screening |
| Boland, 2022 (15) | Recommendations | Cancer | Colorectal | Secondary | Genomics | PTEN-Hamartoma Tumor Syndrome genetic testing | Personalised screening |
| Boland, 2022 (15) | Recommendations | Cancer | Breast | Secondary | Genomics | PTEN-Hamartoma Tumor Syndrome genetic testing | Personalised screening |
| Boland, 2022 (15) | Recommendations | Cancer | Uterine | Secondary | Genomics | PTEN-Hamartoma Tumor Syndrome genetic testing | Personalised screening |
| Boland, 2022 (15) | Recommendations | Cancer | Thyroid | Secondary | Genomics | PTEN-Hamartoma Tumor Syndrome genetic testing | Personalised screening |
| Boland, 2022 (15) | Recommendations | Cancer | Renal | Secondary | Genomics | PTEN-Hamartoma Tumor Syndrome genetic testing | Personalised screening |
| Boland, 2022 (15) | Recommendations | Cancer | Melanoma | Secondary | Genomics | PTEN-Hamartoma Tumor Syndrome genetic testing | Personalised screening |
| Bonache, 2018 (16) | Cohort study | Cancer | Breast | Secondary | Genomics | Multi gene panel | Personalised screening |
| Bonache, 2018 (16) | Cohort study | Cancer | Ovarian | Primary | Genomics | Multi gene panel | Prophylactic surgery |
| Bonache, 2018 (16) | Cohort study | Cancer | Colorectal | Secondary | Genomics | Multi gene panel | Personalised screening |
| Bonache, 2018 (16) | Cohort study | Cancer | Breast | Primary | Genomics | Multi gene panel | Prophylactic surgery |
| Bousman, 2023 (17) | Guidelines | Neuropsychiatric disorder | Depression disorders | Tertiary | Pharmacogenomics | CYP2D6, CYP2C19, CYP2B6, SLC6A4, HTR2A genotyping | Personalised therapy |
| Božina, 2020 (18) | Recommendations | CVD | MACE (PCI) | Tertiary | Pharmacogenomics | VKORC1, SLCO1B1, CYP2C19, CYP2D6, CYP2C9, CYP4F2 genotyping | Personalised therapy |
| Braamskamp, 2017 (19) | Clinical trial | CVD | MACE (FH) | Primary | Genetics | FH genetic testing | Preventive therapy |
| Brouwer, 2022 (20) | Guidelines | Neuropsychiatric disorder | Depression disorders | Tertiary | Pharmacogenomics | CYP2C19, CYP2D6 genotyping | Personalised therapy |
| C. Celis-Morales, 2017 (21) | Clinical trial | Metabolic conditions | Type 2 diabetes | Primary | Genetics | MTHFR, FTO, TCF7L2, APOE e4, FADS1 testing | Lifestyle changes |
| C. Celis-Morales, 2017 (21) | Clinical trial | Metabolic conditions | Obesity | Primary | Genetics | MTHFR, FTO, TCF7L2, APOE e4, FADS1 testing | Lifestyle changes |
| C. Celis-Morales, 2017 (21) | Clinical trial | CVD | MACE | Primary | Genetics | MTHFR, FTO, TCF7L2, APOE e4, FADS1 testing | Lifestyle changes |
| Cardoso, 2019 (22) | Guidelines | Cancer | Breast | Tertiary | Genetics | Oncotype DX | Target therapy |
| Cardoso, 2019 (22) | Guidelines | Cancer | Breast | Tertiary | Genetics | MammaPrint | Target therapy |
| Cardoso, 2019 (22) | Guidelines | Cancer | Breast | Tertiary | Genetics | Prosigna | Target therapy |
| Cardoso, 2019 (22) | Guidelines | Cancer | Breast | Tertiary | Genetics | Endopredict | Target therapy |
| Cardoso, 2019 (22) | Guidelines | Cancer | Breast | Tertiary | Genetics | Brest Cancer Index | Target therapy |
| Cavallari, 2017 (23) | Clinical trial | CVD | MACE | Tertiary | Pharmacogenomics | CYP2C19 genotyping | Personalised therapy |
| Cavallari, 2018 (24) | Clinical trial | CVD | MACE | Tertiary | Pharmacogenomics | CYP2C19 genotyping | Personalised therapy |
| Claassens, 2019 (25) | Clinical trial | CVD | MACE | Tertiary | Pharmacogenomics | CYP2C19 genotyping | Personalised therapy |
| Cooper-DeHoff, 2022 (26) | Guidelines | CVD | MACE (hypercholesterolemia) | Primary | Pharmacogenomics | SLCO1B1, ABCG2, CYP2C9 genotyping | Personalised therapy |
| Cooper-DeHoff, 2022 (26) | Guidelines | CVD | MACE | Tertiary | Pharmacogenomics | SLCO1B1, ABCG2, CYP2C9 genotyping | Personalised therapy |
| Corcoran, 2018 (27) | Clinical trial | Cancer | Colorectal | Tertiary | Genetics | BRAF v600e testing | Target therapy |
| Crosbie, 2019 (28) | Guidelines | Cancer | Ovarian | Primary | Genetics | Lynch Syndrome genetic testing | Prophylactic surgery |
| Crosbie, 2019 (28) | Guidelines | Cancer | Uterine | Primary | Genetics | Lynch Syndrome genetic testing | Prophylactic surgery |
| Crowder, 2022 (29) | Clinical trial | Cancer | Melanoma | Primary | Genetics | MC1R testing | Lifestyle changes |
| Cuchel, 2023 (30) | Recommendations | CVD | MACE (FH) | Primary | Genetics | LDLR, APOB, PCSK9, LDLRAP1 testing | Lifestyle changes |
| Daly, 2021 (31) | Guidelines | Cancer | Breast | Primary | Genetics | BRCA1/2 testing | Lifestyle changes |
| Daly, 2021 (31) | Guidelines | Cancer | Breast | Primary | Genetics | BRCA1/2 testing | Prophylactic surgery |
| Daly, 2021 (31) | Guidelines | Cancer | Ovarian | Primary | Genetics | BRCA1/2 testing | Lifestyle changes |
| Daly, 2021 (31) | Guidelines | Cancer | Ovarian | Primary | Genetics | BRCA1/2 testing | Prophylactic surgery |
| Daly, 2021 (31) | Guidelines | Cancer | Breast | Secondary | Genetics | BRCA1/2 testing | Personalised screening |
| Daly, 2021 (31) | Guidelines | Cancer | Breast | Primary | Genetics | Li-Fraumeni Syndrome genetic testing | Lifestyle changes |
| Daly, 2021 (31) | Guidelines | Cancer | Breast | Primary | Genetics | Li-Fraumeni Syndrome genetic testing | Prophylactic surgery |
| Daly, 2021 (31) | Guidelines | Cancer | Breast | Secondary | Genetics | Li-Fraumeni Syndrome genetic testing | Personalised screening |
| Daly, 2021 (31) | Guidelines | Cancer | Colorectal | Secondary | Genetics | Li-Fraumeni Syndrome genetic testing | Personalised screening |
| Daniel, 2020 (32) | Clinical trial | CVD | MACE (FH) | Primary | Genetics | FH genetic testing | Preventive therapy |
| De Bono, 2017 (33) | Clinical trial | Cancer | Ovarian | Tertiary | Genetics | BRCA1/2 testing | Target therapy |
| De Bono, 2017 (33) | Clinical trial | Cancer | Pancreatic | Tertiary | Genetics | BRCA1/2 testing | Target therapy |
| De Bono, 2017 (33) | Clinical trial | Cancer | Lung (SC) | Tertiary | Genetics | BRCA1/2 testing | Target therapy |
| De Falco, 2020 (34) | Cohort study | Cancer | Gastric | Tertiary | Genomics | FoundationOne CDx, a tool for CGP through NGS panel, was used to evaluate various tumour samples and understand the mutation - ERBB2 + MSI/TMB | Target therapy |
| De Falco, 2020 (34) | Cohort study | Cancer | Colorectal | Tertiary | Genomics | FoundationOne CDx, a tool for CGP through NGS panel, was used to evaluate various tumour samples and understand the mutation KRAS + AXL | Target therapy |
| De Falco, 2020 (34) | Cohort study | Cancer | Pancreatic | Tertiary | Genomics | FoundationOne CDx, a tool for CGP through NGS panel, was used to evaluate various tumour samples and understand the mutation ETV6-NTRK3 fusion | Target therapy |
| De Falco, 2020 (34) | Cohort study | Cancer | Cholangiocarcinoma | Tertiary | Genomics | FoundationOne CDx, a tool for CGP through NGS panel, was used to evaluate various tumour samples and understand the mutation IDH R132C | Target therapy |
| Dean, 2017 (35) | Guidelines | CVD | AF | Tertiary | Pharmacogenomics | CYP2D6 genotyping | Personalised therapy |
| Ditsch, 2019 (36) | Recommendations | Cancer | Breast | Primary | Genetics | BRCA1/2 testing | Prophylactic surgery |
| Ditsch, 2019 (36) | Recommendations | Cancer | Breast | Primary | Genetics | BRCA1/2 testing | Preventive therapy |
| Ditsch, 2019 (36) | Recommendations | Cancer | Breast | Secondary | Genetics | BRCA1/2 testing | Personalised screening |
| Ditsch, 2019 (36) | Recommendations | Cancer | Ovarian | Secondary | Genetics | BRCA1/2 testing | Personalised screening |
| Ditsch, 2019 (36) | Recommendations | Cancer | Ovarian | Primary | Genetics | BRCA1/2 testing | Prophylactic surgery |
| Ditsch, 2019 (36) | Recommendations | Cancer | Ovarian | Primary | Genetics | BRCA1/2 testing | Preventive therapy |
| Ditsch, 2019 (36) | Recommendations | Cancer | Breast | Tertiary | Genetics | BRCA1/2 testing | Target therapy |
| Ditsch, 2019 (36) | Recommendations | Cancer | Ovarian | Tertiary | Genetics | BRCA1/2 testing | Target therapy |
| Elliott, 2017 (37) | Clinical trial | CVD | MACE | Tertiary | Pharmacogenomics | All common and rare variants with known clinical significance at analytical sensitivity and specificity greater than 99% | Personalised therapy |
| Elliott, 2017 (37) | Clinical trial | CVD | MACE (hypertension) | Primary | Pharmacogenomics | All common and rare variants with known clinical significance at analytical sensitivity and specificity greater than 99% | Personalised therapy |
| Elliott, 2017 (37) | Clinical trial | Others | COPD | Tertiary | Pharmacogenomics | All common and rare variants with known clinical significance at analytical sensitivity and specificity greater than 99% | Personalised therapy |
| Elliott, 2017 (37) | Clinical trial | Others | Gastroesophageal reflux | Tertiary | Pharmacogenomics | All common and rare variants with known clinical significance at analytical sensitivity and specificity greater than 99% | Personalised therapy |
| Elliott, 2017 (37) | Clinical trial | CVD | AF | Tertiary | Pharmacogenomics | All common and rare variants with known clinical significance at analytical sensitivity and specificity greater than 99% | Personalised therapy |
| Elliott, 2017 (37) | Clinical trial | Metabolic conditions | Type 2 diabetes | Tertiary | Pharmacogenomics | All common and rare variants with known clinical significance at analytical sensitivity and specificity greater than 99% | Personalised therapy |
| Elliott, 2017 (37) | Clinical trial | Neuropsychiatric disorder | Alzheimer | Tertiary | Pharmacogenomics | All common and rare variants with known clinical significance at analytical sensitivity and specificity greater than 99% | Personalised therapy |
| Elliott, 2017 (37) | Clinical trial | Neuropsychiatric disorder | Epilepsy | Tertiary | Pharmacogenomics | All common and rare variants with known clinical significance at analytical sensitivity and specificity greater than 99% | Personalised therapy |
| Elliott, 2017 (37) | Clinical trial | CVD | MACE | Tertiary | Pharmacogenomics | All common and rare variants with known clinical significance at analytical sensitivity and specificity greater than 99% | Personalised therapy |
| Elliott, 2017 (37) | Clinical trial | Others | Hypothyroidism | Tertiary | Pharmacogenomics | All common and rare variants with known clinical significance at analytical sensitivity and specificity greater than 99% | Personalised therapy |
| Elliott, 2017 (37) | Clinical trial | Neuropsychiatric disorder | Depression disorders | Tertiary | Pharmacogenomics | All common and rare variants with known clinical significance at analytical sensitivity and specificity greater than 99% | Personalised therapy |
| Elliott, 2017 (37) | Clinical trial | Neuropsychiatric disorder | Dementia | Tertiary | Pharmacogenomics | All common and rare variants with known clinical significance at analytical sensitivity and specificity greater than 99% | Personalised therapy |
| Elliott, 2017 (37) | Clinical trial | Others | Eye macular degeneration | Tertiary | Pharmacogenomics | All common and rare variants with known clinical significance at analytical sensitivity and specificity greater than 99% | Personalised therapy |
| Elliott, 2017 (37) | Clinical trial | Cancer | Lung | Tertiary | Pharmacogenomics | All common and rare variants with known clinical significance at analytical sensitivity and specificity greater than 99% | Personalised therapy |
| Elliott, 2017 (37) | Clinical trial | Metabolic conditions | Obesity | Tertiary | Pharmacogenomics | All common and rare variants with known clinical significance at analytical sensitivity and specificity greater than 99% | Personalised therapy |
| Evron, 2018 (38) | Clinical trial | Cancer | Breast | Tertiary | Genetics | BRCA1/2 testing | Target therapy |
| Fabrizio, 2022 (39) | Clinical trial | Others | Eye macular degeneration | Primary | Genetics | ARMS2, CFH, IL8, VEGFA, TIMP3, SLC16A8, COL8A1, RAD51B testing | Lifestyle changes |
| Fabrizio, 2022 (39) | Clinical trial | Others | Eye macular degeneration | Secondary | Genetics | ARMS2, CFH, IL8, VEGFA, TIMP3, SLC16A8, COL8A1, RAD51B testing | Personalised screening |
| Flaherty, 2020 (40) | Clinical trial | Cancer | Breast | Tertiary | Genomics | Multi gene panel | Target therapy |
| Flaherty, 2020 (40) | Clinical trial | Cancer | Colorectal | Tertiary | Genomics | Multi gene panel | Target therapy |
| Flaherty, 2020 (40) | Clinical trial | Cancer | Prostate | Tertiary | Genomics | Multi gene panel | Target therapy |
| Flaherty, 2020 (40) | Clinical trial | Cancer | Lung | Tertiary | Genomics | Multi gene panel | Target therapy |
| Frebourg, 2020 (41) | Clinical trial | Cancer | Breast | Secondary | Genetics | TP53 testing | Personalised screening |
| Frebourg, 2020 (41) | Clinical trial | Cancer | Soft-tissue sarcoma | Secondary | Genetics | TP53 testing | Personalised screening |
| Frebourg, 2020 (41) | Clinical trial | Cancer | Osteosarcoma | Secondary | Genetics | TP53 testing | Personalised screening |
| Frebourg, 2020 (41) | Clinical trial | Cancer | Snc | Secondary | Genetics | TP53 testing | Personalised screening |
| Frebourg, 2020 (41) | Clinical trial | Cancer | Endocrine | Secondary | Genetics | TP53 testing | Personalised screening |
| Furniss, 2021 (42) | Clinical trial | Cancer | Pancreatic | Primary | Genomics | 30-gene hereditary cancer panel | Lifestyle changes |
| Furniss, 2021 (42) | Clinical trial | Cancer | Pancreatic | Secondary | Genomics | 30-gene hereditary cancer panel | Genetic counselling and personalised screening of healthy relatives |
| Gage, 2017 (43) | Clinical trial | CVD | MACE | Tertiary | Pharmacogenomics | VKORC1-1639G>A, CYP2C9*2, CYP2C9*3, CYP4F2 V433M genotyping | Personalised therapy |
| Gammal, 2023 (44) | Clinical trial | Metabolic conditions | Gout | Tertiary | Pharmacogenomics | G6PD genotyping | Personalised therapy |
| Ganti, 2024 (45) | Clinical trial | Cancer | Lung (SC) | Tertiary | Genetics | PDL-1 testing | Target therapy |
| Garon, 2019 (46) | Clinical trial | Cancer | Lung (NSCLC) | Tertiary | Genetics | Genetic testing | Target therapy |
| Gilligan, 2024 (47) | Clinical trial | Cancer | Testicular | Tertiary | Genetics | MSI/MMR, TMB testing | Target therapy |
| Giudicessi, 2017 (48) | Clinical trial | CVD | Cardiomyopathies | Secondary | Genetics | Genetic testing for suspected patients | Genetic counselling and personalised screening of healthy relatives |
| Giudicessi, 2017 (48) | Clinical trial | CVD | Cardiomyopathies | Tertiary | Genetics | Genetic testing for cardiomyopathies | Personalised therapy |
| Giudicessi, 2017 (48) | Clinical trial | CVD | MACE (channelopathies) | Primary | Genetics | CVDs genetic testing | Preventive therapy |
| Giudicessi, 2017 (48) | Clinical trial | CVD | MACE (FH) | Primary | Genetics | FH genetic testing | Lifestyle changes |
| Giudicessi, 2017 (48) | Clinical trial | CVD | MACE (FH) | Primary | Genetics | FH genetic testing | Preventive therapy |
| Goetz, 2018 (49) | Clinical trial | Cancer | Breast | Tertiary | Pharmacogenomics | CYP2D6 genotyping | Personalised therapy |
| Goggins, 2020 (50) | Clinical trial | Cancer | Pancreatic | Secondary | Genetics | Peutz Jeghers Syndrome genetic testing | Personalised screening |
| Goggins, 2020 (50) | Clinical trial | Cancer | Pancreatic | Primary | Genetics | Peutz Jeghers Syndrome genetic testing | Prophylactic surgery |
| Gori, 2019 (51) | Recommendations | Cancer | Ovarian | Tertiary | Genetics | BRCA1/2 testing | Target therapy |
| Gori, 2019 (51) | Recommendations | Cancer | Breast | Secondary | Genetics | BRCA1/2 testing | Genetic counselling and personalised screening of healthy relatives |
| Gori, 2019 (51) | Recommendations | Cancer | Breast | Tertiary | Genetics | BRCA1/2 testing | Target therapy |
| Gori, 2019 (51) | Recommendations | Cancer | Ovarian | Primary | Genetics | BRCA1/2 testing | Risk-reduction strategies |
| Gori, 2019 (51) | Recommendations | Cancer | Breast | Primary | Genomics | BRCA1/2 testing | Risk-reduction strategies |
| Haddad, 2024 (52) | Guidelines | Cancer | Thyroid | Tertiary | Genetics | BRAF, NTRK, ALK, RET, MSI, dMMR, TMB testing | Target therapy |
| Hanson, 2023 (53) | Guidelines | Cancer | Breast | Secondary | Genetics | CHEK2 testing | Personalised screening |
| Harada-Shiba, 2023 (54) | Guidelines | CVD | MACE (FH) | Primary | Genetics | FH genomic testing | Preventive therapy |
| Haverfield, 2021 (55) | Cohort study | Cancer | Gastrointestinal | Secondary | Genomics | Multi gene panel | Personalised screening |
| Haverfield, 2021 (55) | Cohort study | Cancer | Breast | Secondary | Genomics | Multi gene panel | Personalised screening |
| Haverfield, 2021 (55) | Cohort study | Cancer | Ovarian | Secondary | Genomics | Multi gene panel | Personalised screening |
| Haverfield, 2021 (55) | Cohort study | Cancer | Melanoma | Secondary | Genomics | Multi gene panel | Personalised screening |
| Haverfield, 2021 (55) | Cohort study | Cancer | Endocrine | Secondary | Genomics | Multi gene panel | Personalised screening |
| Haverfield, 2021 (55) | Cohort study | Cancer | Prostate | Secondary | Genomics | Multi gene panel | Personalised screening |
| Haverfield, 2021 (55) | Cohort study | Cancer | SNC | Secondary | Genomics | Multi gene panel | Personalised screening |
| Haverfield, 2021 (55) | Cohort study | Cancer | Renal | Secondary | Genomics | Multi gene panel | Personalised screening |
| Henricks, 2018 (56) | Cohort study | Cancer | Colorectal | Tertiary | Pharmacogenomics | DPYD genotyping | Personalised therapy |
| Herzig, 2017 (57) | Guidelines | Cancer | Colorectal | Primary | Genetics | FAP test (APC mutation testing) | Prophylactic surgery |
| Herzig, 2017 (57) | Guidelines | Cancer | Colorectal | Secondary | Genetics | FAP test (APC mutation testing) | Personalised screening |
| Holter, 2022 (58) | Recommendations | Cancer | Colorectal | Tertiary | Genetics | Lynch Syndrome genetic testing | Target therapy |
| Holter, 2022 (58) | Recommendations | Cancer | Colorectal | Secondary | Genetics | Lynch Syndrome genetic testing | Personalised screening |
| Horne, 2020 (59) | Clinical trial | Metabolic conditions | Obesity | Primary | Genomics | UCP1, FTO, TCF7L2, APOA2, PPARγ 2, MC4R genotyping | Lifestyle changes |
| Hulot, 2020 (60) | Cohort study | CVD | MACE | Tertiary | Pharmacogenomics | CYP2C9 genotyping | Personalised therapy |
| Hulshof, 2023 (61) | Guidelines | Cancer | Colorectal | Tertiary | Pharmacogenomics | UGT1A1 genotyping | Personalised therapy |
| Johnson, 2017 (62) | Guidelines | CVD | MACE (thromboembolism) | Primary | Pharmacogenomics | CYP2C9, VKORC1, CYP4F2 genotyping | Personalised therapy |
| Karnes, 2021 (63) | Guidelines | Neuropsychiatric disorder | Epilepsy | Tertiary | Pharmacogenomics | CYP2C9, HLA-B genotyping | Personalised therapy |
| Kastelein, 2017 (64) | Clinical trial | CVD | MACE (FH) | Primary | Genetics | FH genetic testing | Preventive therapy |
| Kim, 2021 (65) | Clinical trial | Cancer | Breast | Primary | Genomics | PRS | Preventive therapy |
| Kinnamon, 2023 (66) | Clinical trial | CVD | Dilated cardiomyopathy | Primary | - | - | Lifestyle changes |
| Kopetz, 2019 (67) | Clinical trial | Cancer | Colorectal | Tertiary | Genetics | BRAF v600e testing | Target therapy |
| Kopetz, 2019 (68) | Clinical trial | Cancer | Colorectal | Tertiary | Genomics | NGS | Target therapy |
| Kopetz, 2019 (68) | Clinical trial | Cancer | Breast | Tertiary | Genomics | NGS | Target therapy |
| Kopetz, 2019 (68) | Clinical trial | Cancer | Lung | Tertiary | Genomics | NGS | Target therapy |
| Kopetz, 2019 (68) | Clinical trial | Cancer | Sarcoma | Tertiary | Genomics | NGS | Target therapy |
| Kopetz, 2019 (68) | Clinical trial | Cancer | Melanoma | Tertiary | Genomics | NGS | Target therapy |
| Kopetz, 2019 (68) | Clinical trial | Cancer | Ovarian | Tertiary | Genomics | NGS | Target therapy |
| Kratz, 2017 (69) | Clinical trial | Cancer | Breast | Secondary | Genetics | Li-Fraumeni Syndrome genetic testing | Personalised screening |
| Kristeleit, 2017 (70) | Clinical trial | Cancer | Ovarian | Tertiary | Genetics | BRCA1/2 testing | Target therapy |
| Lee, 2022 (71) | Clinical trial | CVD | MACE | Tertiary | Pharmacogenomics | CYP2C19 genotyping | Personalised therapy |
| Leitsalu, 2021 (72) | Clinical trial | Cancer | Breast | Primary | Genetics | BRCA1/2 testing | Prophylactic surgery |
| Leitsalu, 2021 (72) | Cohort study | Cancer | Breast | Secondary | Genetics | BRCA1/2 testing | Personalised screening |
| Lunenburg, 2020 (73) | Guidelines | Cancer | Breast | Tertiary | Pharmacogenomics | DPYD genotyping | Personalised therapy |
| Lunenburg, 2020 (73) | Guidelines | Cancer | Melanoma | Tertiary | Pharmacogenomics | DPYD genotyping | Personalised therapy |
| Lunenburg, 2020 (73) | Guidelines | Cancer | Colorectal | Tertiary | Pharmacogenomics | DPYD genotyping | Personalised therapy |
| Lunenburg, 2020 (73) | Guidelines | Cancer | Gastric | Tertiary | Pharmacogenomics | DPYD genotyping | Personalised therapy |
| Mach, 2020 (74) | Guidelines | CVD | MACE (FH) | Primary | Genetics | FH genetic testing | Lifestyle changes |
| Mach, 2020 (74) | Guidelines | CVD | MACE (FH) | Primary | Genetics | FH genetic testing | Preventive therapy |
| Maxwell, 2022 (75) | Clinical trial | Metabolic conditions | Type 2 diabetes | Primary | Genetics | Genetic testing | Lifestyle changes |
| Nabors, 2024 (76) | Clinical trial | Cancer | SNC | Tertiary | Genetics | BRAF V600e, MEK, NTRK testing | Target therapy |
| NCI, 2020 (77) | Clinical trial | Cancer | Breast | Primary | Genetics | BRCA1/2 testing | Prophylactic surgery |
| NCI, 2020 (77) | Clinical trial | Cancer | Breast | Secondary | Genetics | BRCA1/2 testing | Personalised screening |
| NCI, 2020 (77) | Clinical trial | Cancer | Breast | Tertiary | Genetics | BRCA1/2 testing | Target therapy |
| NICE, 2023 (78) | Clinical trial | Cancer | Breast | Tertiary | Genomics | EndoPredict | Target therapy |
| NICE, 2023 (78) | Clinical trial | Cancer | Breast | Tertiary | Genomics | EPClin score | Target therapy |
| NICE, 2023 (78) | Guidelines | Cancer | Breast | Tertiary | Genomics | MammaPrint | Target therapy |
| NICE, 2023 (78) | Guidelines | Cancer | Breast | Tertiary | Genomics | Oncotype DX | Target therapy |
| NICE, 2023 (78) | Guidelines | Cancer | Breast | Tertiary | Genomics | Prosigna | Target therapy |
| Nitz, 2017 (79) | Clinical trial | Cancer | Breast | Tertiary | Genomics | Oncotype DX | Target therapy |
| Notarangelo, 2018 (80) | Clinical trial | CVD | MACE | Tertiary | Pharmacogenomics | ABCB1, CYP2C19*2, CYP2C19*17 genotyping | Personalised therapy |
| Oslin, 2022 (81) | Clinical trial | Neuropsychiatric disorder | Depression disorders | Tertiary | Pharmacogenomics | CYP1A, CYP2B6, CYP2C19, CYP2C9, CYP3A4, CYP2D6, UGT1A4, UGT2B15, SLC6A4, HTR2A, HLA-B*1502, HLA-A*3101 genotyping | Personalised therapy |
| Parker, 2020 (82) | Clinical trial | Cancer | Prostate | Tertiary | Genetics | BRCA1/2 testing | Target therapy |
| Pereira, 2020 (83) | Clinical trial | CVD | MACE | Tertiary | Pharmacogenomics | CYP2C19 genotyping | Personalised therapy |
| Phillips, 2018 (84) | Clinical trial | Neuropsychiatric disorder | Epilepsy | Tertiary | Pharmacogenomics | HLA-B*15:02, HLA-A*31:01 genotyping | Personalised therapy |
| Planchard, 2018 (85) | Clinical trial | Cancer | Lung (NSCLC) | Tertiary | Genetics | EGFR, ALK, ROS1, BRAF testing | Target therapy |
| Raal, 2020 (86) | Clinical trial | CVD | MACE (FH) | Primary | Pharmacogenomics | ANGPTL3 genotyping | Personalised therapy |
| Raal, 2020 (87) | Clinical trial | CVD | MACE (FH) | Primary | Genetics | FH genetic testing | Preventive therapy |
| Rex, 2017 (88) | Recommendations | Cancer | Colorectal | Secondary | Genetics | Lynch Syndrome genetic testing | Personalised screening |
| Rhiem, 2022 (89) | Recommendations | Cancer | Breast | Secondary | Genomics | TrueRisk gene panel | Personalised screening |
| Ridker, 2018 (90) | Clinical trial | CVD | MACE (FH) | Primary | Genetics | FH genetic testing | Preventive therapy |
| Riely, 2024 (91) | Clinical trial | Cancer | Lung (NSCLC) | Tertiary | Genetics | PDL-1, EGFR, ALK, ERBB2, KRAS p.G12C mutations, METex14 skipping, NTRK1/2/3 fusions, RET rearrangements, ROS1 rearrangements testing | Target therapy |
| Roke, 2017 (92) | Clinical trial | Metabolic conditions | Multiple | Primary | Genomics | FADS1 genotyping | Lifestyle changes |
| Russo, 2022 (93) | Clinical trial | Cancer | Breast | Tertiary | Genetics | BRCA1/2 testing | Target therapy |
| Russo, 2022 (93) | Clinical trial | Cancer | Breast | Secondary | Genetics | BRCA1/2 testing | Genetic counselling and personalised screening of healthy relatives |
| Russo, 2022 (93) | Clinical trial | Cancer | Ovarian | Tertiary | Genetics | BRCA1/2 testing | Target therapy |
| Russo, 2022 (93) | Clinical trial | Cancer | Pancreatic | Tertiary | Genetics | BRCA1/2 testing | Target therapy |
| Russo, 2022 (93) | Recommendations | Cancer | Prostate | Tertiary | Genetics | BRCA1/2 testing | Target therapy |
| Russo, 2022 (93) | Recommendations | Cancer | Pancreatic | Secondary | Genetics | BRCA1/2 testing | Genetic counselling and personalised screening of healthy relatives |
| Russo, 2022 (93) | Recommendations | Cancer | Prostate | Secondary | Genetics | BRCA1/2 testing | Genetic counselling and personalised screening of healthy relatives |
| Russo, 2022 (93) | Recommendations | Cancer | Ovarian | Primary | Genetics | BRCA1/2 testing | Risk-reduction strategies |
| Russo, 2022 (93) | Recommendations | Cancer | Pancreatic | Primary | Genetics | BRCA1/2 testing | Risk-reduction strategies |
| Russo, 2022 (93) | Recommendations | Cancer | Prostate | Primary | Genetics | BRCA1/2 testing | Risk-reduction strategies |
| Russo, 2022 (93) | Recommendations | Cancer | Breast | Primary | Genetics | BRCA1/2 testing | Risk-reduction strategies |
| Russo, 2022(93) | Clinical trial | Cancer | Ovarian | Secondary | Genetics | BRCA1/2 testing | Genetic counselling and personalised screening of healthy relatives |
| Sánchez, 2019 (94) | Clinical trial | Cancer | Cholangiocarcinoma | Tertiary | Genomics | Multiple 73 genes | Target therapy |
| Sánchez, 2019 (94) | Clinical trial | Cancer | Pancreatic | Tertiary | Genomics | Multiple 73 genes | Target therapy |
| Sánchez, 2019 (94) | Clinical trial | Cancer | Hepatic | Tertiary | Genomics | Multiple 73 genes | Target therapy |
| Sánchez, 2019 (94) | Clinical trial | Cancer | Breast | Tertiary | Genomics | Multiple 73 genes | Target therapy |
| Sánchez, 2019 (94) | Clinical trial | Cancer | Lung | Tertiary | Genomics | Multiple 73 genes | Target therapy |
| Sánchez, 2019 (94) | Clinical trial | Cancer | Colorectal | Tertiary | Genomics | Multiple 73 genes | Target therapy |
| Sánchez, 2019 (94) | Clinical trial | Cancer | Prostate | Tertiary | Genomics | Multiple 73 genes | Target therapy |
| Sánchez, 2019 (94) | Cohort study | Cancer | Ovarian | Tertiary | Genomics | Multiple 73 genes | Target therapy |
| Santos, 2020 (95) | Clinical trial | CVD | MACE (FH) | Primary | Genetics | FH genetic testing | Preventive therapy |
| Schaeffer, 2024 (96) | Clinical trial | Cancer | Prostate | Tertiary | Genetics | BRCA1/2 testing | Target therapy |
| Seppala, 2021 (97) | Guidelines | Cancer | Colorectal | Secondary | Genetics | Lynch Syndrome genetic testing | Personalised screening |
| Smit, 2021 (98) | Clinical trial | Cancer | Melanoma | Primary | Genomics | PRS | Lifestyle changes |
| Sparano, 2018 (99) | Clinical trial | Cancer | Breast | Tertiary | Genetics | 21-gene expression assay | Target therapy |
| Sparks, 2018 (100) | Clinical trial | Others | Rheumatoid arthritis | Primary | Genomics | Genotype (HLA-DRB1) + autoantibody (RF/CCP) | Lifestyle changes |
| Stoffel, 2019 (101) | Guidelines | Cancer | Pancreatic | Secondary | Genetics | Germline mutations detections in advanced pancreatic cancer patients and genetic counselling and screening for healthy relatives | Personalised screening |
| Sturm, 2018 (102) | Recommendations | CVD | MACE (FH) | Primary | Genetics | PCSK9, ApoB, LDLR testing | Preventive therapy |
| Sturm, 2018 (102) | Recommendations | CVD | MACE (FH) | Secondary | Genetics | PCSK9, ApoB, LDLR testing | Genetic counselling and personalised screening of healthy relatives |
| Swetter, 2024 (103) | Guidelines | Cancer | Melanoma | Tertiary | Genetics | BRAF v600e, KIT, PDL-1 testing | Target therapy |
| Swisher, 2017 (104) | Clinical trial | Cancer | Ovarian | Tertiary | Genetics | BRCA1/2 testing | Target therapy |
| Tempero, 2024 (105) | Guidelines | Cancer | Pancreatic | Tertiary | Genetics | BRAF V600E, NTRK, RET, MSIH testing | Target therapy |
| Tischkowitz, 2020 (106) | Guidelines | Cancer | Breast | Secondary | Genetics | PTEN testing | Personalised screening |
| Tischkowitz, 2020 (106) | Guidelines | Cancer | Thyroid | Secondary | Genetics | PTEN testing | Personalised screening |
| Tischkowitz, 2021 (107) | Guidelines | Cancer | Breast | Secondary | Genetics | PALB2 testing | Personalised screening |
| Tuteja, 2020 (108) | Clinical trial | CVD | MACE | Tertiary | Pharmacogenomics | CYP2C9 genotyping | Personalised therapy |
| Tutrone, 2020 (109) | Clinical trial | Cancer | Prostate | Secondary | Genetics | ExoDx Prostate(IntelliScore) (EPI) test | Personalised screening |
| Van Cutsem, 2019 (110) | Clinical trial | Cancer | Colorectal | Tertiary | Genetics | BRAF v600e testing | Target therapy |
| Vassy, 2017 (111) | Clinical trial | CVD | Hypertrophic cardiomyopathy | Secondary | Genomics | WGS | Genetic counselling and personalised screening of healthy relatives |
| Vassy, 2020 (112) | Clinical trial | CVD | MACE (atherosclerotic cardiovascular disease) | Primary | Pharmacogenomics | SLCO1B1 genotyping | Personalised therapy |
| Viigimaa, 2022 (113) | Clinical trial | CVD | MACE | Primary | Genomics | PRS | Lifestyle changes |
| von Minckwitz, 2017 (114) | Clinical trial | Cancer | Breast | Tertiary | Immunoistochemistry | HER2 testing | Target therapy |
| Wagner, 2021 (115) | Guidelines | Cancer | Colorectal | Secondary | Genetics | Peutz Jeghers Syndrome genetic testing | Personalised screening |
| Wagner, 2021 (115) | Guidelines | Cancer | Pancreatic | Secondary | Genetics | Peutz Jeghers Syndrome genetic testing | Personalised screening |
| Weiss, 2017 (116) | Clinical trial | Cancer | Lung (SC) | Tertiary | Genetics | Genetic testing | Target therapy |
| Weiss, 2021 (117) | Guidelines | Cancer | Colorectal | Secondary | Genetics | FAP1/2 (APC gene testing) | Personalised screening |
| Widen, 2022 (118) | Cohort study | CVD | MACE | Primary | Genomics | PRS | Lifestyle changes |
| Wolever, 2022 (119) | Clinical trial | CVD | MACE | Primary | Genetics | CHD genetic testing | Lifestyle changes |
| Wolever, 2022 (119) | Clinical trial | Metabolic conditions | Type 2 diabetes | Primary | Genetics | T2D genetic testing | Lifestyle changes |
| Xiao, 2022 (120) | Clinical trial | CVD | MACE (hypertension) | Primary | Pharmacogenomics | CYP2D6*10, ADRB1, CYP2C9*3, AGTR1, ACE, CYP3A5*3, NPPA genotyping | Personalised therapy |
| Zheng, 2020 (121) | Clinical trial | CVD | MACE (PCI) | Tertiary | Pharmacogenomics | Testing for MAR | Personalised therapy |

**Acronyms**

ABCB1 = ATP-Binding Cassette Sub-Family B Member 1

ACE = Angiotensin-Converting Enzyme

ADRB1 = Adrenergic Receptor Beta 1

AF = Atrial Fibrillation

AGTR1 = Angiotensin II Receptor Type 1

ALK = Anaplastic Lymphoma Kinase

ANGPTL3 = Angiopoietin-like 3

APO = Apolipoprotein

BRAF = B-Raf proto-oncogene

BRCA1/2 = Breast Cancer 1 and 2

CGP = Comprehensive Genomic Profiling

CHD = Coronary Heart Disease

CHEK2 = Checkpoint Kinase 2

COL8A1 = Collagen Type VIII Alpha 1

COPD = Chronic Obstructive Pulmonary Disease

CVD = Cardiovascular Diseases

CYP = Cytochrome P450

DPYD = Dihydropyrimidine Dehydrogenase

EGFR = Epidermal Growth Factor Receptor

ERBB2 = Human Epidermal Growth Factor Receptor 2

ESR1 = Estrogen Receptor 1

FADS1 = Fatty Acid Desaturase 1

FAP = Familial Adenomatous Polyposis

FH = Familial Hypercholesterolemia

FTO = Fat Mass and Obesity Associated gene

G6PD = Glucose-6-Phosphate Dehydrogenase

HER2 = Human Epidermal Growth Factor Receptor 2

HLA = Human Leukocyte Antigens

HR = Hormone Receptor

HTR2A = 5-Hydroxytryptamine Receptor 2A

IDH = Isocitrate Dehydrogenase

IHC = Immunohistochemistry

KRAS = Kirsten Rat Sarcoma viral oncogene homolog

LDLR = Low-Density Lipoprotein Receptor

MACE = Major Adverse Cardiovascular Events (infarction, stroke)

MAR = Maximum Aggregation Rate

MC1R = Melanocortin 1 Receptor

MC4R = Melanocortin 4 Receptor

MET = Mesenchymal Epithelial Transition factor

MMR = Mismatch Repair

MSI = Microsatellite Instability

MTHFR = Methylenetetrahydrofolate Reductase

NGS = Next-Generation Sequencing

NSCLC = Non-Small-Cell Lung Cancer

NTRK = Neurotrophic Receptor Tyrosine Kinase

PALB2 = Partner and Localizer of BRCA2

PCI = Percutaneous Coronary Intervention

PCSK9 = Proprotein Convertase Subtilisin/Kexin Type 9

PDL-1 = Programmed Death-Ligand 1

PIK3CA = Phosphoinositide-3-Kinase Catalytic Subunit Alpha

PJS = Peutz Jeger Syndrome

PRS = Polygenic Risk Score

RAS = Rat Sarcoma

RET = REarranged during Transfection

ROS1 = Gene C-Ros Oncogene 1

SC = Small-Cell

SLC = Solute Carrier

SLCO= Solute Carrier Organic Anion Transporter

T2D = Type 2 Diabetes

TCF7L2 = Transcription Factor 7 Like 2

TMB = Tumor Mutational Burden

TP53 = Tumor Protein P53

UCP1 = Uncoupling Protein 1

UGT1A1 = Uridine Diphosphate Glucuronosyltransferase 1A1

VHL = Von Hippel-Lindau syndrome

VKORC1 = Vitamin K Epoxide Reductase Complex 1

WGS = Whole Genome Sequencing

**References**

1. Abraham J, Chew H, Dwyer MA, Kumar R, Gradishar WJ, Elias AD, et al. Breast Cancer, Version 4.2023 - NCCN Guidelines. JNCCN Journal of the National Comprehensive Cancer Network. 2023 Jun 1;21(6):594–608.

2. Abu-Rustum NR, Campos SM, Yashar CM, Gaffney DK, Gaillard S, Giuntoli RI, et al. Cervical Cancer, Version 3.2024 - NCCN Guidelines [Internet]. 2024. Available from: https://www.accessdata.fda.gov/drugsatfda_docs/label/2024/125514s147lbl.pdf

3. Abu-Rustum NR, Yashar CM, Arend R, Barber E, Bradley K, Brooks R. Uterine Neoplasms, Version 2.2024 - NCCN Guidelines [Internet]. 2024. Available from: www.nccn.org/patients

4. Aguirre AJ, Nowak JA, Camarda ND, Moffitt RA, Ghazani AA, Hazar-Rethinam M, et al. Real-time genomic characterization of advanced pancreatic cancer to enable precision medicine. Cancer Discov [Internet]. 2018 Sep 1 [cited 2024 Aug 1];8(9):1096. Available from: /pmc/articles/PMC6192263/

5. Ajani JA, D’Amico TA, Bentrem DJ, Chao J, Cooke D, Corvera C, et al. Gastric Cancer, Version 2.2022, NCCN Clinical Practice Guidelines in Oncology. Journal of the National Comprehensive Cancer Network [Internet]. 2022 Feb 1 [cited 2024 Aug 1];20(2):167–92. Available from: https://jnccn.org/view/journals/jnccn/20/2/article-p167.xml

6. Alonso R, Muñiz-Grijalvo O, Díaz-Díaz JL, Zambón D, de Andrés R, Arroyo-Olivares R, et al. Efficacy of PCSK9 inhibitors in the treatment of heterozygous familial hypercholesterolemia: A clinical practice experience. J Clin Lipidol [Internet]. 2021 May 7 [cited 2024 Aug 1];15(4):584–92. Available from: https://europepmc.org/article/MED/34052174

7. Alver M, Palover M, Saar A, Läll K, Zekavat SM, Tõnisson N, et al. Recall by genotype and cascade screening for familial hypercholesterolemia in a population-based biobank from Estonia. Genetics in Medicine 2018 21:5 [Internet]. 2018 Oct 1 [cited 2024 Aug 1];21(5):1173–80. Available from: https://www.nature.com/articles/s41436-018-0311-2

8. Amstutz U, Henricks LM, Offer SM, Barbarino J, Schellens JHM, Swen JJ, et al. Clinical Pharmacogenetics Implementation Consortium (CPIC) Guideline for Dihydropyrimidine Dehydrogenase Genotype and Fluoropyrimidine Dosing: 2017 Update HHS Public Access. Clin Pharmacol Ther [Internet]. 2018 [cited 2024 Aug 1];103(2):210–6. Available from: https://cpicpgx.org/guidelines/

9. Andre F, Ismaila N, Henry NL, Somerfield MR, Bast RC, Barlow W, et al. Use of Biomarkers to Guide Decisions on Adjuvant Systemic Therapy for Women With Early-Stage Invasive Breast Cancer: ASCO Clinical Practice Guideline Update-Integration of Results From TAILORx. J Clin Oncol [Internet]. 2019 Aug 1 [cited 2024 Aug 1];37(22):1956–64. Available from: https://pubmed.ncbi.nlm.nih.gov/31150316/

10. Armstrong DK, Alvarez RD, Backes FJ, Barroilhet L, Behbakht K, Berchuck A. Ovarian Cancer, Version 3.2024 - NCCN Guidelines [Internet]. 2024. Available from: www.nccn.org/patients

11. Benson AB, Venook AP, Adam M, Chang GJ, Chen YJ, Ciombor KK, et al. Colon Cancer, Version 4.2024 - NCCN Guidelines [Internet]. 2024. Available from: https://www.nccn.org/home/member-

12. Beunk L, Nijenhuis M, Soree B, de Boer-Veger NJ, Buunk AM, Guchelaar HJ, et al. Dutch Pharmacogenetics Working Group (DPWG) guideline for the gene-drug interaction between CYP2D6, CYP3A4 and CYP1A2 and antipsychotics. Eur J Hum Genet [Internet]. 2024 Mar 1 [cited 2024 Aug 1];32(3):278–85. Available from: https://pubmed.ncbi.nlm.nih.gov/37002327/

13. Louise M Binderup M, Smerdel M, Borgwadt L, Beck Nielsen SS, Madsen MG, Møller HU, et al. von Hippel-Lindau disease: Updated guideline for diagnosis and surveillance. Eur J Med Genet. 2022 Aug 1;65(8):104538.

14. Birnbaum RA, Horton BH, Gidding SS, Brenman LM, Macapinlac BA, Avins AL. Closing the gap: Identification and management of familial hypercholesterolemia in an integrated healthcare delivery system. J Clin Lipidol [Internet]. 2021 Mar 1 [cited 2024 Aug 1];15(2):347–57. Available from: https://pubmed.ncbi.nlm.nih.gov/33583725/

15. Boland CR, Idos GE, Durno C, Giardiello FM, Anderson JC, Burke CA, et al. Diagnosis and Management of Cancer Risk in the Gastrointestinal Hamartomatous Polyposis Syndromes: Recommendations From the US Multi-Society Task Force on Colorectal Cancer. Gastroenterology [Internet]. 2022 Jun 1 [cited 2024 Aug 1];162(7):2063–85. Available from: https://pubmed.ncbi.nlm.nih.gov/35487791/

16. Bonache S, Esteban I, Moles-Fernández A, Tenés A, Duran-Lozano L, Montalban G, et al. Multigene panel testing beyond BRCA1/2 in breast/ovarian cancer Spanish families and clinical actionability of findings. J Cancer Res Clin Oncol [Internet]. 2018 Dec 1 [cited 2024 Aug 1];144(12):2495–513. Available from: https://pubmed.ncbi.nlm.nih.gov/30306255/

17. Bousman CA, Stevenson JM, Ramsey LB, Sangkuhl K, Hicks JK, Strawn JR, et al. Clinical Pharmacogenetics Implementation Consortium (CPIC) Guideline for CYP2D6, CYP2C19, CYP2B6, SLC6A4, and HTR2A Genotypes and Serotonin Reuptake Inhibitor Antidepressants. Clin Pharmacol Ther. 2023 Jul 1;114(1):51–68.

18. Božina N, Kirhmajer MV, Šimičević L, Ganoci L, Skvrce NM, Domjanović IK, et al. Use of pharmacogenomics in elderly patients treated for cardiovascular diseases. Croat Med J [Internet]. 2020 Apr 1 [cited 2024 Aug 1];61(2):147–58. Available from: https://pubmed.ncbi.nlm.nih.gov/32378381/

19. Braamskamp MJAM, Langslet G, Mccrindle BW, Cassiman D, Francis GA, Gagne C, et al. Effect of Rosuvastatin on Carotid Intima-Media Thickness in Children With Heterozygous Familial Hypercholesterolemia: The CHARON Study (Hypercholesterolemia in Children and Adolescents Taking Rosuvastatin Open Label). Circulation [Internet]. 2017 Jun 7 [cited 2024 Aug 1];136(4):359–66. Available from: https://europepmc.org/article/MED/28592434

20. Brouwer JMJL, Nijenhuis M, Soree B, Guchelaar HJ, Swen JJ, van Schaik RHN, et al. Dutch Pharmacogenetics Working Group (DPWG) guideline for the gene-drug interaction between CYP2C19 and CYP2D6 and SSRIs. Eur J Hum Genet [Internet]. 2022 Oct 1 [cited 2024 Aug 1];30(10):1114–20. Available from: https://pubmed.ncbi.nlm.nih.gov/34782755/

21. Celis-Morales C, Livingstone KM, Marsaux CFM, Macready AL, Fallaize R, O’Donovan CB, et al. Effect of personalized nutrition on health-related behaviour change: evidence from the Food4Me European randomized controlled trial. Int J Epidemiol [Internet]. 2017 [cited 2024 Aug 1];46(2):578–88. Available from: https://pubmed.ncbi.nlm.nih.gov/27524815/

22. Cardoso F, Kyriakides S, Ohno S, Penault-Llorca F, Poortmans P, Rubio IT, et al. Early breast cancer: ESMO Clinical Practice Guidelines for diagnosis, treatment and follow-up†. Annals of Oncology [Internet]. 2019 Aug 1 [cited 2024 Aug 1];30(8):1194–220. Available from: http://www.annalsofoncology.org/article/S0923753419312876/fulltext

23. Cavallari LH, Lee CR, Beitelshees AL, Cooper-DeHoff RM, Duarte JD, Voora D, et al. Multisite Investigation of Outcomes With Implementation of CYP2C19 Genotype-Guided Antiplatelet Therapy After Percutaneous Coronary Intervention. JACC Cardiovasc Interv [Internet]. 2017 Nov 1 [cited 2024 Aug 1];11(2):181–91. Available from: https://europepmc.org/articles/PMC5775044

24. Cavallari LH, Franchi F, Rollini F, Been L, Rivas A, Agarwal M, et al. Clinical implementation of rapid CYP2C19 genotyping to guide antiplatelet therapy after percutaneous coronary intervention Journal of Translational Medicine. J Transl Med [Internet]. 2018 [cited 2024 Aug 1];16:92. Available from: https://doi.org/10.1186/s12967-018-1469-8

25. Claassens DMF, Vos GJA, Bergmeijer TO, Hermanides RS, van ’t Hof AWJ, van der Harst P, et al. A Genotype-Guided Strategy for Oral P2Y 12 Inhibitors in Primary PCI . New England Journal of Medicine. 2019 Oct 24;381(17):1621–31.

26. Cooper-Dehoff RM, Niemi M, Ramsey LB, Luzum JA, Katriina Tarkiainen E, Straka RJ, et al. The Clinical Pharmacogenetics Implementation Consortium Guideline for SLCO1B1, ABCG2, and CYP2C9 genotypes and Statin-Associated Musculoskeletal Symptoms. CLINICAL PHARMACOLOGY & THERAPEUTICS | VOLUME [Internet]. 2022 [cited 2024 Aug 1];111. Available from: www.cpt-journal.com

27. Corcoran RB, Andre T, Atreya CE, Schellens JHM, Yoshino T, Bendell JC, et al. Research article combined BRAF, EGFR, and MEK inhibition in patients with BRAF V600E -mutant colorectal cancer. Cancer Discov [Internet]. 2018 Apr 1 [cited 2024 Aug 1];8(4):428–43. Available from: /cancerdiscovery/article/8/4/428/9620/Combined-BRAF-EGFR-and-MEK-Inhibition-in-Patients

28. Crosbie EJ, Ryan NAJ, Arends MJ, Bosse T, Burn J, Cornes JM, et al. The Manchester International Consensus Group recommendations for the management of gynecological cancers in Lynch syndrome. Genet Med [Internet]. 2019 Oct 1 [cited 2024 Aug 1];21(10):2390–400. Available from: https://pubmed.ncbi.nlm.nih.gov/30918358/

29. Crowder SL, Buro AW, Lacson JCA, Kim Y, Sutton SK, Roetzheim RG, et al. Retention and evaluation of precision and generic prevention materials for melanoma: a qualitative study comparing young adults and adults. Cancer Prev Res (Phila) [Internet]. 2022 Aug 8 [cited 2024 Aug 1];15(8):533. Available from: /pmc/articles/PMC9357111/

30. Cuchel M, Raal FJ, Hegele RA, Al-Rasadi K, Arca M, Averna M, et al. 2023 Update on European Atherosclerosis Society Consensus Statement on Homozygous Familial Hypercholesterolaemia: new treatments and clinical guidance. Eur Heart J [Internet]. 2023 Jul 1 [cited 2024 Aug 1];44(25):2277–91. Available from: https://pubmed.ncbi.nlm.nih.gov/37130090/

31. Daly MB, Pal T, Berry MP, Buys SS, Dickson P, Domchek SM, et al. Genetic/Familial High-Risk Assessment: Breast, Ovarian, and Pancreatic, Version 2.2021, NCCN Clinical Practice Guidelines in Oncology. Journal of the National Comprehensive Cancer Network [Internet]. 2021 Jan 6 [cited 2024 Aug 1];19(1):77–102. Available from: https://jnccn.org/view/journals/jnccn/19/1/article-p77.xml

32. Daniels S, Caprio S, Chaudhari U, Manvelian G, Baccara-Dinet MT, Brunet A, et al. PCSK9 inhibition with alirocumab in pediatric patients with heterozygous familial hypercholesterolemia: The ODYSSEY KIDS study. J Clin Lipidol [Internet]. 2020 Mar 28 [cited 2024 Aug 1];14(3):322-330.e5. Available from: https://europepmc.org/article/MED/32331936

33. de Bono J, Ramanathan RK, Mina L, Chugh R, Glaspy J, Rafii S, et al. Phase I, dose-escalation, two-part trial of the PARP inhibitor talazoparib in patients with advanced germline BRCA1/2 mutations and selected sporadic cancers. Cancer Discov. 2017;7(6):620–9.

34. De Falco V, Poliero L, Vitello PP, Ciardiello D, Vitale P, Zanaletti N, et al. Feasibility of next-generation sequencing in clinical practice: results of a pilot study in the Department of Precision Medicine at the University of Campania “Luigi Vanvitelli.” ESMO Open [Internet]. 2020 Mar 30 [cited 2024 Aug 1];5(2). Available from: https://pubmed.ncbi.nlm.nih.gov/32234730/

35. Dean L. Propafenone Therapy and CYP2D6 Genotype [Internet]. 2017. Available from: https://www.ncbi.nlm.nih.gov/books/

36. Ditsch N, Untch M, Thill M, Müller V, Janni W, Albert US, et al. AGO Recommendations for the Diagnosis and Treatment of Patients with Early Breast Cancer: Update 2019. Breast Care [Internet]. 2019 Aug 1 [cited 2024 Aug 1];14(4):224. Available from: /pmc/articles/PMC6751475/

37. Elliott LS, Henderson JC, Neradilek MB, Moyer NA, Ashcraft KC, Thirumaran RK. Clinical impact of pharmacogenetic profiling with a clinical decision support tool in polypharmacy home health patients: A prospective pilot randomized controlled trial. 2017 [cited 2024 Aug 1]; Available from: http://genelex.com/

38. Evron E, David MA Ben, Goldberg H, Fried G, Kaufman B, Catane R, et al. Phase II national clinical trial of prophylactic irradiation to the contralateral breast for BRCA mutation carriers treated for early breast cancer (EBC). https://doi.org/101200/JCO20183615_suppl514 [Internet]. 2018 Jun 1 [cited 2024 Aug 1];36(15_suppl):514–514. Available from: https://ascopubs.org/doi/10.1200/JCO.2018.36.15_suppl.514

39. Fabrizio C, Termine A, Caputo V, Megalizzi D, Zampatti S, Falsini B, et al. WARE: Wet AMD Risk-Evaluation Tool as a Clinical Decision-Support System Integrating Genetic and Non-Genetic Factors. J Pers Med [Internet]. 2022 Jul 1 [cited 2024 Aug 1];12(7). Available from: https://pubmed.ncbi.nlm.nih.gov/35887531/

40. Flaherty KT, Gray R, Chen A, Li S, Patton D, Hamilton SR, et al. The Molecular Analysis for Therapy Choice (NCI-MATCH) Trial: Lessons for Genomic Trial Design. J Natl Cancer Inst [Internet]. 2020 Oct 1 [cited 2024 Aug 1];112(10):1021–9. Available from: https://pubmed.ncbi.nlm.nih.gov/31922567/

41. Frebourg T, Bajalica Lagercrantz S, Oliveira C, Magenheim R, Evans DG, Hoogerbrugge N, et al. Guidelines for the Li–Fraumeni and heritable TP53-related cancer syndromes. European Journal of Human Genetics 2020 28:10 [Internet]. 2020 May 26 [cited 2024 Aug 1];28(10):1379–86. Available from: https://www.nature.com/articles/s41431-020-0638-4

42. Furniss CS, Yurgelun MB, Ukaegbu C, Constantinou PE, Lafferty CC, Talcove-Berko ER, et al. Novel Models of Genetic Education and Testing for Pancreatic Cancer Interception: Preliminary Results from the GENERATE Study. Cancer Prev Res (Phila) [Internet]. 2021 Nov 1 [cited 2024 Aug 1];14(11):1021–32. Available from: https://pubmed.ncbi.nlm.nih.gov/34625409/

43. Gage BF, Bass AR, Lin H, Woller SC, Stevens SM, Al-Hammadi N, et al. Effect of Genotype-Guided Warfarin Dosing on Clinical Events and Anticoagulation Control Among Patients Undergoing Hip or Knee Arthroplasty: The GIFT Randomized Clinical Trial. JAMA [Internet]. 2017 Sep 26 [cited 2024 Aug 1];318(12):1115–24. Available from: https://jamanetwork.com/journals/jama/fullarticle/2654820

44. Gammal RS, Pirmohamed M, Somogyi AA, Morris SA, Formea CM, Elchynski AL, et al. Expanded Clinical Pharmacogenetics Implementation Consortium Guideline for Medication Use in the Context of G6PD Genotype. CLINICAL PHARMACOLOGY & THERAPEUTICS | VOLUME [Internet]. 2023 [cited 2024 Aug 1];113. Available from: https://ascpt.onlinelibrary.wiley.com/doi/10.1002/cpt.2735,

45. Ganti AKP, Loo BW, Chair V, Badiyan S, Bassetti M, Bestvina C, et al. Small Cell Lung Cancer, Version 3.2024 - NCCN Guidelines [Internet]. 2024. Available from: https://www.nccn.org/home/member-

46. Garon EB, Hellmann MD, Rizvi NA, Carcereny E, Leighl NB, Ahn MJ, et al. Five-Year Overall Survival for Patients With Advanced Non‒Small-Cell Lung Cancer Treated With Pembrolizumab: Results From the Phase I KEYNOTE-001 Study. J Clin Oncol [Internet]. 2019 Oct 1 [cited 2024 Aug 1];37(28):2518–27. Available from: https://pubmed.ncbi.nlm.nih.gov/31154919/

47. Gilligan T, Adra N, Bagrodia A, Costa D, Drakaki A, Emamekhoo H, et al. Testicular Cancer, Version 1.2024 - NCCN Guidelines [Internet]. 2024. Available from: https://www.nccn.org/home/member-

48. Giudicessi JR, Kullo IJ, Ackerman MJ. Precision Cardiovascular Medicine: State of Genetic Testing. Mayo Clin Proc [Internet]. 2017 Apr 1 [cited 2024 Aug 1];92(4):642–62. Available from: http://www.mayoclinicproceedings.org/article/S0025619617300897/fulltext

49. Goetz MP, Sangkuhl K, Guchelaar HJ, Schwab M, Province M, Whirl-Carrillo M, et al. Clinical Pharmacogenetics Implementation Consortium (CPIC) Guideline for CYP2D6 and Tamoxifen Therapy. Clin Pharmacol Ther. 2018 May 1;103(5):770–7.

50. Goggins M, Overbeek KA, Brand R, Syngal S, Del Chiaro M, Bartsch DK, et al. Management of patients with increased risk for familial pancreatic cancer: updated recommendations from the International Cancer of the Pancreas Screening (CAPS) Consortium. Gut [Internet]. 2020 Jan 1 [cited 2024 Aug 1];69(1):7. Available from: /pmc/articles/PMC7295005/

51. Gori S, Barberis M, Bella MA, Buttitta F, Capoluongo E, Carrera P, et al. Recommendations for the implementation of BRCA testing in ovarian cancer patients and their relatives. Crit Rev Oncol Hematol [Internet]. 2019 Aug 1 [cited 2024 Aug 1];140:67–72. Available from: https://pubmed.ncbi.nlm.nih.gov/31176273/

52. Haddad RI, Agosto Salgado S, Applewhite M, Blomain E, Lamki Busaidy N, Campbell M, et al. Thyroid Carcinoma,Version 3.2024 - NCCN Guidelines [Internet]. 2024. Available from: https://www.ncbi.nlm.nih.gov/

53. Hanson H, Astiazaran-Symonds E, Amendola LM, Balmaña J, Foulkes WD, James P, et al. Management of individuals with germline pathogenic/likely pathogenic variants in CHEK2: A clinical practice resource of the American College of Medical Genetics and Genomics (ACMG). Genet Med [Internet]. 2023 Oct 1 [cited 2024 Aug 1];25(10). Available from: https://pubmed.ncbi.nlm.nih.gov/37490054/

54. Harada-Shiba M, Arai H, Ohmura H, Okazaki H, Sugiyama D, Tada H, et al. Guidelines for the Diagnosis and Treatment of Adult Familial Hypercholesterolemia 2022. Vol. 30, Journal of Atherosclerosis and Thrombosis. Japan Atherosclerosis Society; 2023. p. 558–86.

55. Haverfield E V., Esplin ED, Aguilar SJ, Hatchell KE, Ormond KE, Hanson-Kahn A, et al. Physician-directed genetic screening to evaluate personal risk for medically actionable disorders: a large multi-center cohort study. BMC Med [Internet]. 2021 Dec 1 [cited 2024 Aug 1];19(1). Available from: https://pubmed.ncbi.nlm.nih.gov/34404389/

56. Henricks LM, Lunenburg CATC, de Man FM, Meulendijks D, Frederix GWJ, Kienhuis E, et al. DPYD genotype-guided dose individualisation of fluoropyrimidine therapy in patients with cancer: a prospective safety analysis. Lancet Oncol [Internet]. 2018 Nov 1 [cited 2024 Aug 1];19(11):1459–67. Available from: https://pubmed.ncbi.nlm.nih.gov/30348537/

57. Herzig D, Hardimann K, Weiser M, Yu N, Paquette I, Feingold DL, et al. Clinical Practice Guidelines for the Management of Inherited Polyposis Syndromes HHS Public Access. Dis Colon Rectum. 2017;60(9):881–94.

58. Holter S, Hall MJ, Hampel H, Jasperson K, Kupfer SS, Larsen Haidle J, et al. Risk assessment and genetic counseling for Lynch syndrome - Practice resource of the National Society of Genetic Counselors and the Collaborative Group of the Americas on Inherited Gastrointestinal Cancer. J Genet Couns [Internet]. 2022 Jun 1 [cited 2024 Aug 1];31(3):568–83. Available from: https://pubmed.ncbi.nlm.nih.gov/35001450/

59. Horne J, Gilliland J, O’Connor C, Seabrook J, Madill J. Enhanced long-term dietary change and adherence in a nutrigenomics-guided lifestyle intervention compared to a population-based (GLB/DPP) lifestyle intervention for weight management: results from the NOW randomised controlled trial. BMJ Nutr Prev Health [Internet]. 2020 Jun [cited 2024 Aug 1];3(1):49–59. Available from: https://pubmed.ncbi.nlm.nih.gov/33235971/

60. Hulot JS, Chevalier B, Belle L, Cayla G, Khalife K, Funck F, et al. Routine CYP2C19 Genotyping to Adjust Thienopyridine Treatment After Primary PCI for STEMI: Results of the GIANT Study. JACC Cardiovasc Interv [Internet]. 2020 Mar 9 [cited 2024 Aug 1];13(5):621–30. Available from: https://pubmed.ncbi.nlm.nih.gov/32139220/

61. Hulshof EC, Deenen MJ, Nijenhuis M, Soree B, de Boer-Veger NJ, Buunk AM, et al. Dutch pharmacogenetics working group (DPWG) guideline for the gene-drug interaction between UGT1A1 and irinotecan. Eur J Hum Genet [Internet]. 2023 Sep 1 [cited 2024 Aug 1];31(9):982–7. Available from: https://pubmed.ncbi.nlm.nih.gov/36443464/

62. Johnson JA, Caudle KE, Gong L, Whirl-Carrillo M, Stein CM, Scott SA, et al. Clinical Pharmacogenetics Implementation Consortium (CPIC) Guideline for Pharmacogenetics-Guided Warfarin Dosing: 2017 Update. Clin Pharmacol Ther. 2017 Sep 1;102(3):397–404.

63. Karnes JH, Rettie AE, Somogyi AA, Huddart R, Fohner AE, Formea CM, et al. Clinical Pharmacogenetics Implementation Consortium (CPIC) Guideline for CYP2C9 and HLA-B Genotypes and Phenytoin Dosing: 2020 Update. 2021 [cited 2024 Aug 2];109:302. Available from: https://www.pharm

64. Kastelein JJP, Hovingh GK, Langslet G, Baccara-Dinet MT, Gipe DA, Chaudhari U, et al. Efficacy and safety of the proprotein convertase subtilisin/kexin type 9 monoclonal antibody alirocumab vs placebo in patients with heterozygous familial hypercholesterolemia. J Clin Lipidol [Internet]. 2017 Jan 1 [cited 2024 Aug 1];11(1):195-203.e4. Available from: http://www.lipidjournal.com/article/S1933287416304512/fulltext

65. Kim JO, Schaid DJ, Vachon CM, Cooke A, Couch FJ, Kim CA, et al. Impact of personalized genetic breast cancer risk estimation with polygenic risk scores on preventive endocrine therapy intention and uptake. Cancer Prevention Research [Internet]. 2021 Feb 1 [cited 2024 Aug 1];14(2):175–84. Available from: /cancerpreventionresearch/article/14/2/175/47393/Impact-of-Personalized-Genetic-Breast-Cancer-Risk

66. Kinnamon DD, Jordan E, Haas GJ, Hofmeyer M, Kransdorf E, Ewald GA, et al. Effectiveness of the Family Heart Talk Communication Tool in Improving Family Member Screening for Dilated Cardiomyopathy: Results of a Randomized Trial. Circulation [Internet]. 2023 Apr 25 [cited 2024 Aug 1];147(17):1281–90. Available from: https://pubmed.ncbi.nlm.nih.gov/36938756/

67. Kopetz S, Grothey A, Yaeger R, Van Cutsem E, Desai J, Yoshino T, et al. Encorafenib, Binimetinib, and Cetuximab in BRAF V600E–Mutated Colorectal Cancer . New England Journal of Medicine [Internet]. 2019 Oct 24 [cited 2024 Aug 1];381(17):1632–43. Available from: https://www.nejm.org/doi/full/10.1056/NEJMoa1908075

68. Kopetz S, Mills Shaw KR, Lee JJ, Zhang J, Litzenburger B, Holla V, et al. Use of a Targeted Exome Next-Generation Sequencing Panel Offers Therapeutic Opportunity and Clinical Benefit in a Subset of Patients With Advanced Cancers. JCO Precis Oncol [Internet]. 2019 Dec 8 [cited 2024 Aug 1];(3):1–14. Available from: https://ascopubs.org/doi/10.1200/PO.18.00213

69. Kratz CP, Achatz MI, Brugieres L, Frebourg T, Garber JE, Greer MLC, et al. Cancer Screening Recommendations for Individuals with Li-Fraumeni Syndrome. Clin Cancer Res [Internet]. 2017 Jun 1 [cited 2024 Aug 1];23(11):e38–45. Available from: https://pubmed.ncbi.nlm.nih.gov/28572266/

70. Kristeleit R, Shapiro GI, Burris HA, Oza AM, LoRusso P, Patel MR, et al. A Phase I-II Study of the Oral PARP Inhibitor Rucaparib in Patients with Germline BRCA1/2-Mutated Ovarian Carcinoma or Other Solid Tumors. Clin Cancer Res [Internet]. 2017 Aug 1 [cited 2024 Aug 1];23(15):4095–106. Available from: https://pubmed.ncbi.nlm.nih.gov/28264872/

71. Lee CR, Luzum JA, Sangkuhl K, Gammal RS, Sabatine MS, Stein CM, et al. Clinical Pharmacogenetics Implementation Consortium Guideline for CYP2C19 Genotype and Clopidogrel Therapy: 2022 Update. Clin Pharmacol Ther. 2022 Nov 1;112(5):959–67.

72. Leitsalu L, Palover M, Sikka TT, Reigo A, Kals M, Pärn K, et al. Genotype-first approach to the detection of hereditary breast and ovarian cancer risk, and effects of risk disclosure to biobank participants. European Journal of Human Genetics [Internet]. 2021 Mar 1 [cited 2024 Aug 1];29(3):471. Available from: /pmc/articles/PMC7940387/

73. Lunenburg CATC, van der Wouden CH, Nijenhuis M, Crommentuijn-van Rhenen MH, de Boer-Veger NJ, Buunk AM, et al. Dutch Pharmacogenetics Working Group (DPWG) guideline for the gene-drug interaction of DPYD and fluoropyrimidines. Eur J Hum Genet [Internet]. 2020 Apr 1 [cited 2024 Aug 1];28(4):508–17. Available from: https://pubmed.ncbi.nlm.nih.gov/31745289/

74. Mach F, Baigent C, Catapano AL, Koskinas KC, Casula M, Badimon L, et al. 2019 ESC/EAS Guidelines for the management of dyslipidaemias: lipid modification to reduce cardiovascular risk: The Task Force for the management of dyslipidaemias of the European Society of Cardiology (ESC) and European Atherosclerosis Society (EAS). Eur Heart J [Internet]. 2020 Jan 1 [cited 2024 Aug 1];41(1):111–88. Available from: https://dx.doi.org/10.1093/eurheartj/ehz455

75. Maxwell TJ, Franks PW, Kahn SE, Knowler WC, Mather KJ, Florez JC, et al. Quantitative trait loci, G×E and G×G for glycemic traits: response to metformin and placebo in the Diabetes Prevention Program (DPP). J Hum Genet [Internet]. 2022 Aug 1 [cited 2024 Aug 1];67(8):465–73. Available from: https://pubmed.ncbi.nlm.nih.gov/35260800/

76. Nabors BL, Portnow J, Baehring J, Bhatia A, Bloch O, Brem S, et al. Central Nervous System Cancers, Version 2.2024 - NCCN Guidelines [Internet]. 2024. Available from: https://www.nccn.org/home/member-

77. BRCA Gene Mutations: Cancer Risk and Genetic Testing Fact Sheet - NCI [Internet]. [cited 2024 Aug 1]. Available from: https://www.cancer.gov/about-cancer/causes-prevention/genetics/brca-fact-sheet

78. NATIONAL INSTITUTE FOR HEALTH AND CARE EXCELLENCE Diagnostics Assessment Programme Tumour profiling tests to guide adjuvant chemotherapy decisions in lymph node-positive early breast cancer (provisional title).

79. Nitz U, Gluz O, Christgen M, Kates RE, Clemens M, Malter W, et al. Reducing chemotherapy use in clinically high-risk, genomically low-risk pN0 and pN1 early breast cancer patients: five-year data from the prospective, randomised phase 3 West German Study Group (WSG) PlanB trial. Breast Cancer Res Treat [Internet]. 2017 Oct 1 [cited 2024 Aug 1];165(3):573. Available from: /pmc/articles/PMC6336763/

80. Notarangelo FM, Maglietta G, Bevilacqua P, Cereda M, Merlini PA, Villani GQ, et al. Pharmacogenomic Approach to Selecting Antiplatelet Therapy in Patients With Acute Coronary Syndromes: The PHARMCLO Trial. J Am Coll Cardiol [Internet]. 2018 May 1 [cited 2024 Aug 1];71(17):1869–77. Available from: https://pubmed.ncbi.nlm.nih.gov/29540324/

81. Oslin DW, Lynch KG, Shih MC, Ingram EP, Wray LO, Chapman SR, et al. Effect of Pharmacogenomic Testing for Drug-Gene Interactions on Medication Selection and Remission of Symptoms in Major Depressive Disorder: The PRIME Care Randomized Clinical Trial. JAMA [Internet]. 2022 Jul 12 [cited 2024 Aug 1];328(2):151–61. Available from: https://pubmed.ncbi.nlm.nih.gov/35819423/

82. Parker C, Castro E, Fizazi K, Heidenreich A, Ost P, Procopio G, et al. Prostate cancer: ESMO Clinical Practice Guidelines for diagnosis, treatment and follow-up†. Annals of Oncology [Internet]. 2020 Sep 1 [cited 2024 Aug 1];31(9):1119–34. Available from: http://www.annalsofoncology.org/article/S0923753420398987/fulltext

83. Pereira NL, Farkouh ME, So D, Lennon R, Geller N, Mathew V, et al. Effect of Genotype-Guided Oral P2Y12 Inhibitor Selection vs Conventional Clopidogrel Therapy on Ischemic Outcomes After Percutaneous Coronary Intervention: The TAILOR-PCI Randomized Clinical Trial. JAMA [Internet]. 2020 Aug 25 [cited 2024 Aug 1];324(8):761–71. Available from: https://pubmed.ncbi.nlm.nih.gov/32840598/

84. Phillips EJ, Sukasem C, Whirl-Carrillo M, Müller DJ, Dunnenberger HM, Chantratita W, et al. Clinical Pharmacogenetics Implementation Consortium Guideline for HLA Genotype and Use of Carbamazepine and Oxcarbazepine: 2017 Update. Clin Pharmacol Ther. 2018 Apr 1;103(4):574–81.

85. Planchard D, Popat S, Kerr K, Novello S, Smit EF, Faivre-Finn C, et al. Metastatic non-small cell lung cancer: ESMO Clinical Practice Guidelines for diagnosis, treatment and follow-up†. Annals of Oncology [Internet]. 2018 Oct 1 [cited 2024 Aug 1];29:iv192–237. Available from: http://www.annalsofoncology.org/article/S0923753419317107/fulltext

86. Raal FJ, Rosenson RS, Reeskamp LF, Hovingh GK, Kastelein JJP, Rubba P, et al. Evinacumab for Homozygous Familial Hypercholesterolemia. N Engl J Med [Internet]. 2020 Aug 20 [cited 2024 Aug 1];383(8):711–20. Available from: https://pubmed.ncbi.nlm.nih.gov/32813947/

87. Raal FJ, Kallend D, Ray KK, Turner T, Koenig W, Wright RS, et al. Inclisiran for the Treatment of Heterozygous Familial Hypercholesterolemia. N Engl J Med [Internet]. 2020 Mar 18 [cited 2024 Aug 1];382(16):1520–30. Available from: https://europepmc.org/article/MED/32197277

88. Rex DK, Boland CR, Dominitz JA, Giardiello FM, Johnson DA, Kaltenbach T, et al. Colorectal Cancer Screening: Recommendations for Physicians and Patients from the U.S. Multi-Society Task Force on Colorectal Cancer. Am J Gastroenterol [Internet]. 2017 Jul 1 [cited 2024 Aug 1];112(7):1016–30. Available from: https://pubmed.ncbi.nlm.nih.gov/28555630/

89. Rhiem K, Auber B, Briest S, Dikow N, Ditsch N, Dragicevic N, et al. Consensus Recommendations of the German Consortium for Hereditary Breast and Ovarian Cancer. Breast Care (Basel) [Internet]. 2022 Apr 1 [cited 2024 Aug 1];17(2):199–207. Available from: https://pubmed.ncbi.nlm.nih.gov/35702495/

90. Ridker PM, Rose LM, Kastelein JJP, Santos RD, Wei C, Revkin J, et al. Cardiovascular event reduction with PCSK9 inhibition among 1578 patients with familial hypercholesterolemia: Results from the SPIRE randomized trials of bococizumab. J Clin Lipidol. 2018 Jul 1;12(4):958–65.

91. Riely GJ, Wood DE, Aisner DL, Akerley W, Bauman JR, Bruno DS, et al. Non-Small Cell Lung Cancer, Version 7.2024 - NCCN Guidelines [Internet]. 2024. Available from: https://www.nccn.org/home/member-

92. Roke K, Walton K, Klingel SL, Harnett A, Subedi S, Haines J, et al. Evaluating Changes in Omega-3 Fatty Acid Intake after Receiving Personal FADS1 Genetic Information: A Randomized Nutrigenetic Intervention. Nutrients [Internet]. 2017 Mar 6 [cited 2024 Aug 1];9(3). Available from: https://pubmed.ncbi.nlm.nih.gov/28272299/

93. Russo A, Incorvaia L, Capoluongo E, Tagliaferri P, Gori S, Cortesi L, et al. Implementation of preventive and predictive BRCA testing in patients with breast, ovarian, pancreatic, and prostate cancer: a position paper of Italian Scientific Societies. ESMO Open [Internet]. 2022 Jun 1 [cited 2024 Aug 1];7(3). Available from: https://pubmed.ncbi.nlm.nih.gov/35597177/

94. Sánchez NS, Kahle MP, Bailey AM, Wathoo C, Balaji K, Demirhan ME, et al. Identification of Actionable Genomic Alterations Using Circulating Cell-Free DNA. JCO Precis Oncol [Internet]. 2019 Dec [cited 2024 Aug 1];3(3):1–10. Available from: https://pubmed.ncbi.nlm.nih.gov/32923868/

95. Santos RD, Ruzza A, Hovingh GK, Wiegman A, Mach F, Kurtz CE, et al. Evolocumab in Pediatric Heterozygous Familial Hypercholesterolemia. N Engl J Med [Internet]. 2020 Aug 29 [cited 2024 Aug 1];383(14):1317–27. Available from: https://europepmc.org/article/MED/32865373

96. Schaeffer EM, Lurie RH, Adra N, An Y, Bitting R, Chapin B, et al. Prostate Cancer, Version 4.2024 - NCCN Guidelines [Internet]. 2024. Available from: https://www.nccn.org/home/

97. Seppälä TT, Latchford A, Negoi I, Sampaio Soares A, Jimenez-Rodriguez R, Evans DG, et al. European guidelines from the EHTG and ESCP for Lynch syndrome: an updated third edition of the Mallorca guidelines based on gene and gender. Br J Surg [Internet]. 2021 May 1 [cited 2024 Aug 1];108(5):484–98. Available from: https://pubmed.ncbi.nlm.nih.gov/34043773/

98. Smit AK, Allen M, Beswick B, Butow P, Dawkins H, Dobbinson SJ, et al. Impact of personal genomic risk information on melanoma prevention behaviors and psychological outcomes: a randomized controlled trial. Genet Med [Internet]. 2021 Dec 1 [cited 2024 Aug 1];23(12):2394–403. Available from: https://pubmed.ncbi.nlm.nih.gov/34385669/

99. Sparano JA, Gray RJ, Makower DF, Pritchard KI, Albain KS, Hayes DF, et al. Adjuvant Chemotherapy Guided by a 21-Gene Expression Assay in Breast Cancer. N Engl J Med [Internet]. 2018 Jul 12 [cited 2024 Aug 1];379(2):111–21. Available from: https://pubmed.ncbi.nlm.nih.gov/29860917/

100. Sparks JA, Iversen MD, Yu Z, Triedman NA, Prado MG, Miller Kroouze R, et al. Disclosure of Personalized Rheumatoid Arthritis Risk Using Genetics, Biomarkers, and Lifestyle Factors to Motivate Health Behavior Improvements: A Randomized Controlled Trial. Arthritis Care Res (Hoboken) [Internet]. 2018 Jun 1 [cited 2024 Aug 1];70(6):823–33. Available from: https://pubmed.ncbi.nlm.nih.gov/29024454/

101. Stoffel EM, McKernin SE, Khorana AA. Evaluating susceptibility to pancreatic cancer: ASCO clinical practice provisional clinical opinion summary. J Oncol Pract [Internet]. 2019 Feb 1 [cited 2024 Aug 1];15(2):108–11. Available from: https://ascopubs.org/doi/10.1200/JOP.18.00629

102. Sturm AC, Knowles JW, Gidding SS, Ahmad ZS, Ahmed CD, Ballantyne CM, et al. Clinical Genetic Testing for Familial Hypercholesterolemia: JACC Scientific Expert Panel. J Am Coll Cardiol [Internet]. 2018 Aug 7 [cited 2024 Aug 1];72(6):662–80. Available from: https://pubmed.ncbi.nlm.nih.gov/30071997/

103. Swetter SM, Johnson D, Albertini MR, Barker CA, Bateni S, Baumgartner J. Melanoma: Cutaneous, Version 2.2024 - NCCN Guidelines [Internet]. 2024. Available from: www.nccn.org/patients

104. Swisher EM, Lin KK, Oza AM, Scott CL, Giordano H, Sun J, et al. Rucaparib in relapsed, platinum-sensitive high-grade ovarian carcinoma (ARIEL2 Part 1): an international, multicentre, open-label, phase 2 trial. Lancet Oncol [Internet]. 2017 Jan 1 [cited 2024 Aug 1];18(1):75–87. Available from: https://pubmed.ncbi.nlm.nih.gov/27908594/

105. Tempero MA, Malafa MP, Benson III AB, Cardin DB, Gabriela Chiorean E, Hutchinson Cancer Center Jared Christensen FA, et al. Pancreatic Adenocarcinoma, Version 3.2024 - NCCN Guidelines [Internet]. 2024. Available from: https://www.nccn.org/home/member-

106. Tischkowitz M. Cancer Surveillance Guideline for individuals with PTEN hamartoma tumour syndrome Chrystelle Colas 2 • Sjaak Pouwels 3 • Nicoline Hoogerbrugge 4 • PHTS Guideline Development Group • The European Reference Network GENTURIS. European Journal of Human Genetics [Internet]. 2020 [cited 2024 Aug 1];28:1387–93. Available from: https://doi.org/10.1038/s41431-020-0651-7

107. Tischkowitz M, Balmaña J, Foulkes WD, James P, Ngeow J, Schmutzler R, et al. Management of individuals with germline variants in PALB2: a clinical practice resource of the American College of Medical Genetics and Genomics (ACMG). Genetics in Medicine. 2021 Aug 1;23(8):1416–23.

108. Tuteja S, Glick H, Matthai W, Nachamkin I, Nathan A, Monono K, et al. Prospective CYP2C19 Genotyping to Guide Antiplatelet Therapy Following Percutaneous Coronary Intervention: A Pragmatic Randomized Clinical Trial. Circ Genom Precis Med [Internet]. 2020 Feb 1 [cited 2024 Aug 1];13(1):E002640. Available from: https://pubmed.ncbi.nlm.nih.gov/31928229/

109. Tutrone R, Donovan MJ, Torkler P, Tadigotla V, McLain T, Noerholm M, et al. Clinical utility of the exosome based ExoDx Prostate(IntelliScore) EPI test in men presenting for initial Biopsy with a PSA 2-10 ng/mL. Prostate Cancer Prostatic Dis [Internet]. 2020 Dec 1 [cited 2024 Aug 1];23(4):607–14. Available from: https://pubmed.ncbi.nlm.nih.gov/32382078/

110. Van Cutsem E, Huijberts S, Grothey A, Yaeger R, Cuyle PJ, Elez E, et al. Binimetinib, Encorafenib, and Cetuximab Triplet Therapy for Patients With BRAF V600E-Mutant Metastatic Colorectal Cancer: Safety Lead-In Results From the Phase III BEACON Colorectal Cancer Study [Internet]. Vol. 37, J Clin Oncol. 2019. Available from: https://doi.org/10.

111. Vassy JL, Christensen KD, Schonman EF, Blout CL, Robinson JO, Krier JB, et al. The Impact of Whole-Genome Sequencing on the Primary Care and Outcomes of Healthy Adult Patients: A Pilot Randomized Trial. Ann Intern Med [Internet]. 2017 Aug 1 [cited 2024 Aug 1];167(3):159–69. Available from: https://pubmed.ncbi.nlm.nih.gov/28654958/

112. Vassy JL, Michael Gaziano J, Green RC, Ferguson RE, Advani S, Miller SJ, et al. Effect of Pharmacogenetic Testing for Statin Myopathy Risk vs Usual Care on Blood Cholesterol: A Randomized Clinical Trial. JAMA Netw Open [Internet]. 2020 Dec 2 [cited 2024 Aug 1];3(12). Available from: https://pubmed.ncbi.nlm.nih.gov/33270123/

113. Viigimaa M, Jürisson M, Pisarev H, Kalda R, Alavere H, Irs A, et al. Effectiveness and feasibility of cardiovascular disease personalized prevention on high polygenic risk score subjects: a randomized controlled pilot study. European heart journal open [Internet]. 2022 Nov 1 [cited 2024 Aug 1];2(6). Available from: https://pubmed.ncbi.nlm.nih.gov/36600884/

114. von Minckwitz G, Procter M, de Azambuja E, Zardavas D, Benyunes M, Viale G, et al. Adjuvant Pertuzumab and Trastuzumab in Early HER2-Positive Breast Cancer. New England Journal of Medicine. 2017 Jul 13;377(2):122–31.

115. Wagner A, Aretz S, Auranen A, Bruno MJ, Cavestro GM, Crosbie EJ, et al. Clinical Medicine The Management of Peutz-Jeghers Syndrome: European Hereditary Tumour Group (EHTG) Guideline †. J Clin Med. 2021;10:473.

116. Weiss GJ, Byron SA, Aldrich J, Sangal A, Barilla H, Kiefer JA, et al. A prospective pilot study of genome-wide exome and transcriptome profiling in patients with small cell lung cancer progressing after first-line therapy. PLoS One [Internet]. 2017 Jun 1 [cited 2024 Aug 1];12(6). Available from: /pmc/articles/PMC5460863/

117. Weiss JM, Gupta S, Burke CA, Axell L, Chen LM, Chung DC, et al. NCCN Guidelines® Insights: Genetic/Familial High-Risk Assessment: Colorectal, Version 1.2021: Featured Updates to the NCCN Guidelines. Journal of the National Comprehensive Cancer Network [Internet]. 2021 Oct 15 [cited 2024 Aug 1];19(10):1122–32. Available from: https://jnccn.org/view/journals/jnccn/19/10/article-p1122.xml

118. Widén E, Junna N, Ruotsalainen S, Surakka I, Mars N, Ripatti P, et al. How Communicating Polygenic and Clinical Risk for Atherosclerotic Cardiovascular Disease Impacts Health Behavior: an Observational Follow-up Study. Circ Genom Precis Med [Internet]. 2022 Apr 1 [cited 2024 Aug 1];15(2):E003459. Available from: https://pubmed.ncbi.nlm.nih.gov/35130028/

119. Wolever RQ, Yang Q, Maldonado CJ, Armitage NH, Musty MD, Kraus WE, et al. Health coaching and genetic risk testing in primary care: Randomized controlled trial. Health Psychol [Internet]. 2022 [cited 2024 Aug 1];41(10):719–32. Available from: https://pubmed.ncbi.nlm.nih.gov/35587890/

120. Xiao ZL, Yang M, Chen X Bin, Xie XM, Chen MF. Personalized antihypertensive treatment guided by pharmacogenomics in China. Cardiovasc Diagn Ther [Internet]. 2022 Oct 1 [cited 2024 Aug 1];12(5):635–45. Available from: /pmc/articles/PMC9622397/

121. Zheng YY, Wu TT, Yang Y, Hou XG, Gao Y, Chen Y, et al. Personalized antiplatelet therapy guided by a novel detection of platelet aggregation function in stable coronary artery disease patients undergoing percutaneous coronary intervention: a randomized controlled clinical trial. Eur Heart J Cardiovasc Pharmacother [Internet]. 2020 [cited 2024 Aug 1];6(4):211–21. Available from: https://pubmed.ncbi.nlm.nih.gov/31603191/
